# Correction: *In Vivo* Substrates of the Lens Molecular Chaperones αA-Crystallin and αB-Crystallin

**DOI:** 10.1371/journal.pone.0102210

**Published:** 2014-07-01

**Authors:** The PLOS ONE Staff

## Notice of Republication

This paper was republished on June 10, 2014, to include File S1, which was mistakenly omitted from the original publication; the publisher apologizes for the error. In addition, in the last sentence of the Two-week Old aA-R49C Mouse Lenses section, “Spots 5466” was changed to “Spots 5446.”

Please download this article again to view the correct version. The originally published, uncorrected article and the republished, corrected article are provided here for reference.

## Supporting Information

File S1 Originally published, uncorrected article.
(PDF)

File S2 Republished, corrected article.
(PDF)
