# Supplementary material for: In Vivo Substrates of the Lens Molecular Chaperones αA-Crystallin and αB-Crystallin
Source: PLoS One. 2014 Apr 23;9(4):e95507. doi: 10.1371/journal.pone.0095507 (PMC3997384; doi:10.1371/journal.pone.0095507)
Supplement: Table S1 — Analysis of proteins that showed differences in abundance between 2-day-old WT, 14-day-old WT and 2-day-old αA-R49C homozygous mouse lenses. WT, Wild-type. (DOC) [file pone.0095507.s001.doc]

**TABLE S1** **Protein spots that showed a change in abundance between 2-day-old WT, 14-day-old WT and 2-day-old αA-R49C mutant lenses.** WT, Wild-type.

| Spot number | Protein | UNIPROT accession number | MW kDa | Number of assigned spectra | Fold change | |
| --- | --- | --- | --- | --- | --- | --- |
|  |  |  |  |  | WT 2-day-old vs. WT 14-day-old | WT 2-day-old vs. homozygous 2-day-old |
| 2655 | Filensin | A2AMT1 | 74 | 29 | -1.52 | -2.92 |
|  | Calpain 3 | A2AVV5 | 85 | 10 |  |  |
|  | Ezrin | P26040 | 69 | 7 |  |  |
|  | Thimetoligopeptidase | Q8C1A5 | 78 | 6 |  |  |
|  | Phospholipase A2 activating protein | P27612 | 87 | 2 |  |  |
| 2736 | Heat shock cognate 71 kDa protein | P63017 | 71 | 26 | -1.8 | 2.56 |
|  | Stress 70 protein mitochondrial | P38647 | 74 | 8 |  |  |
|  | Heat shock 70 kDa protein 1B | P17879 | 70 | 7 |  |  |
|  | Ras GTPase activating protein-binding protein | P97855 | 52 | 5 |  |  |
|  | Protein RUFY 3 | Q9D394 | 53 | 5 |  |  |
|  | αA-crystallin | Q569M7 | 20 | 2 |  |  |
| 2759 | Filensin | A2AMT1 | 74 | 2 | -2.89 | 3.09 |
|  | Heat shock cognate 71 kDa protein | P63017 | 71 | 2 |  |  |
|  | SET domain-containing protein 3 | Q91WCO | 67 | 2 |  |  |
| 2972 | T-complex protein 1 subunit theta | P42932 | 60 | 15 | -1.19 | 4.16 |
|  | αA-crystallin | Q569M7 | 20 | 10 |  |  |
|  | 60 kDa heat shock protein mitochondria | P63038 | 61 | 4 |  |  |
|  | Tubulin alpha 1B chain | P05213 | 50 | 3 |  |  |
|  | Vimentin | P20152 | 54 | 2 |  |  |
| 3339 | Phakinin | Q6NVD9 | 46 | 45 | -1.22 | -2.92 |
|  | Actin cytoplasmic 1 | P60710 | 42 | 13 |  |  |
|  | Heterogeneous nuclear ribonucleoprotein | Q9Z2X1 | 46 | 7 |  |  |
| 3343 | Phakinin | Q6NVD9 | 46 | 52 | -1.35 | -3.23 |
|  | Actin cytoplasmic 1 | P60710 | 42 | 18 |  |  |
|  | Heterogeneous nuclear ribonucleoprotein | Q9Z2X1 | 46 | 7 |  |  |
|  | Protein disulfide isomerase | Q922R8 | 48 | 2 |  |  |
| 3790 | αA-crystallin | Q569M7 | 20 | 15 | -1.66 | 15.42 |
|  | Creatine kinase B type | Q04447 | 43 | 10 |  |  |
|  | Actin cytoplasmic 1 | P60710 | 42 | 7 |  |  |
|  | Erlin-2 | Q8BFZ9 | 38 | 2 |  |  |
| 3820 | αA-crystallin | Q569M7 | 20 | 8 | 1.77 | -3.87 |
|  | Alpha enolase | P17182 | 47 | 4 |  |  |
|  | βA3/A1-crystallin | Q9QXC6 | 25 | 2 |  |  |
|  | βB1-crystallin | Q9WVJ5 | 28 | 2 |  |  |
| 3929 | αA-crystallin | Q569M7 | 20 | 19 | -1.73 | 27.64 |
|  | Actin cytoplasmic 1 | P60710 | 42 | 3 |  |  |
|  | Creatine kinase B type | Q04447 | 43 | 2 |  |  |
|  | Nucleophosmin | Q61937 | 33 | 2 |  |  |
| 4032 | PDZ containing protein GIPC1 | Q9Z0G0 | 36 | 8 | -1.35 | -3.32 |
|  | αA-crystallin | Q569M7 | 20 | 7 |  |  |
|  | 60S acidic ribosomal protein P0 | P14869 | 34 | 5 |  |  |
| 4346 | 26S proteasome non ATPase regulatory subunit 7 | P26516 | 37 | 2 | 1.01 | 5.68 |
|  | Annexin A1 | P10107 | 39 | 1 (100%) |  |  |
| 4754 | Microtubule-associated protein RP/EB family member | Q61166 | 30 | 2 | -1.59 | 2.9 |
|  | Glyoxylase domain- containing protein 4 | Q9CPV4 | 33 | 2 |  |  |
|  | Tubulin folding factor B | Q9D1E6 |  |  |  |  |
|  | αA-crystallin | Q569M7 | 20 | 1 |  |  |
| 5106 | βB1-crystallin | Q9WVJ5 | 28 | 23 | 1.65 | -2.77 |
|  | βB3-crystallin | Q9JJU9 | 24 | 3 |  |  |
|  | Hemoglobin subunit β-1 | P02088 | 16 | 2 |  |  |
| 5138 | βB1-crystallin | Q9WVJ5 | 28 | 17 | 1.42 | 3.47 |
|  | βB3-crystallin | Q9JJU9 | 24 | 5 |  |  |
|  | αA-crystallin | Q569M7 | 20 | 4 |  |  |
|  | βA3/A1-crystallin | Q9QXC6 | 25 | 2 |  |  |
| 5212 | βB3-crystallin | Q9JJU9 | 24 | 5 | 1.14 | 3.24 |
|  | αA-crystallin | Q569M7 | 20 | 4 |  |  |
|  | Polyubiquitin-B | P0CG49 | 34 | 4 |  |  |
|  | βB1-crystallin | Q9WVJ5 | 28 | 3 |  |  |
|  | ER resident protein | P57759 | 29 | 3 |  |  |
|  | Heat shock protein β-1 | P14602 | 23 | 2 |  |  |
|  | βA3/A1-crystallin | Q9QXC6 | 25 | 2 |  |  |
| 5372 | Phosphoglycerate mutase 2 | O70250 | 29 | 5 | 1.22 | -2.79 |
|  | Coiled-coil-helix-coil-helix | Q9CRB9 | 26 | 2 |  |  |
|  | Proteasome subunit α type 7 | Q9Z240 | 28 | 2 |  |  |
| 5446 | βB2-crystallin | P62696 | 23 | 19 | 14.61 | -1.24 |
|  | βB3-crystallin | Q9JJU9 | 24 | 8 |  |  |
|  | βA3/A1-crystallin | Q9QXC6 | 25 | 7 |  |  |
|  | αA-crystallin | Q569M7 | 20 | 4 |  |  |
|  | αB-crystallin | P23927 | 21 | 2 |  |  |
|  | γS-crystallin | O35486 | 21 | 2 |  |  |
| 5466 | βB2-crystallin | P62696 | 23 | 40 | 23.51 | -1.99 |
|  | βB3-crystallin | Q9JJU9 | 24 | 11 |  |  |
|  | αA-crystallin | Q569M7 | 20 | 4 |  |  |
|  | βA3/A1-crystallin | Q9QXC6 | 25 | 2 |  |  |
| 5513 | βB1-crystallin | Q9WVJ5 | 28 | 20 | 3.01 | 4.54 |
|  | αA-crystallin | Q569M7 | 20 | 8 |  |  |
|  | βA3/A1-crystallin | Q9QXC6 | 25 | 6 |  |  |
|  | βA4-crystallin | Q9JJV0 | 22 | 6 |  |  |
|  | βA2-crystallin | Q9JJV1 | 22 | 5 |  |  |
|  | βS-crystallin | O35486 | 21 | 3 |  |  |
|  | βB2-crystallin | P62696 | 23 | 2 |  |  |
|  | Proteasome subunit β type-4 | P99026 | 29 | 2 |  |  |
| 5552 | αA-crystallin | Q569M7 | 20 | 13 | 2.35 | -4.73 |
|  | βB2-crystallin | P62696 | 23 | 11 |  |  |
|  | βB3-crystallin | Q9JJU9 | 24 | 9 |  |  |
|  | βA3/A1-crystallin | Q9QXC6 | 25 | 7 |  |  |
|  | βA2-crystallin | Q9JJV1 | 22 | 4 |  |  |
|  | βS-crystallin | O35486 | 21 | 3 |  |  |
| 5603 | αA-crystallin | Q569M7 | 20 | 26 | 1.54 | -6.93 |
|  | βS-crystallin | O35486 | 21 | 5 |  |  |
|  | βA2-crystallin | Q9JJV1 | 22 | 2 |  |  |
| 5715 | γD-crystallin | Q6PGI0 | 21 | 13 | 1.9 | -2.65 |
|  | αB-crystallin | P23927 | 20 | 13 |  |  |
|  | γC ins-crystallin | A3RLDS | 21 | 8 |  |  |
|  | βS-crystallin | O35486 | 21 | 4 |  |  |
|  | γB-crystallin | P04344 | 21 | 4 |  |  |
|  | βB3-crystallin | Q9JJU9 | 24 | 4 |  |  |
|  | γA-crystallin | P04345 | 21 | 4 |  |  |
|  | Hemoglobin subunit β-1 | P02088 | 16 | 3 |  |  |
|  | γE-crystallin | Q03740 | 21 | 2 |  |  |
| 5736 | Proteasome subunit β type 5 | O55234 | 29 | 6 | 2.89 | -2.83 |
|  | Histone H2A type 1 | P22752 | 14 | 3 |  |  |
|  | Peroxiredoxin-1 | P35700 | 14 | 3 |  |  |
|  | Glyceraldehyde-3-phosphate dehydrogenase | P16856 | 36 | 1 |  |  |
| 5878 | αA-crystallin | Q569M7 | 20 | 7 | 1.91 | -13.88 |
|  | βB2-crystallin | P62696 | 23 | 2 |  |  |
| 5883 | αA-crystallin | Q569M7 | 20 | 10 | 2.15 | -4.02 |
|  | βA3/A1-crystallin | Q9QXC6 | 25 | 2 |  |  |
| 5963 | αA-crystallin | Q569M7 | 20 | 17 | 1.31 | 13.03 |
| 5987 | αA-crystallin | Q569M7 | 20 | 16 | 1.61 | -9.08 |
|  | βA4-crystallin | Q9JJV0 | 22 | 3 |  |  |
|  | γA-crystallin | P04345 | 21 | 2 |  |  |
| 5990 | αA-crystallin | Q569M7 | 20 | 29 | -1.2 | 14.37 |
| 6001 | αA-crystallin | Q569M7 | 20 | 41 | 1.15 | -4.21 |
| 6036 | αA-crystallin | Q569M7 | 20 | 31 | 1.28 | -18.99 |
| 6057 | αA-crystallin | Q569M7 | 20 | 7 | 1.19 | -3.36 |
|  | βA3/A1-crystallin | Q9QXC6 | 25 | 1 |  |  |
| 6144 | αA-crystallin | Q569M7 | 20 | 30 | 2.06 | 35.73 |
|  | γA-crystallin | P04345 | 21 | 2 |  |  |
|  | γC-crystallin | Q61597 | 21 | 2 |  |  |
| 6290 | αA-crystallin | Q569M7 | 20 | 10 | -1.04 | 15.9 |
|  | Grifin | Q9D1U0 | 16 | 2 |  |  |
| 6300 | γC-crystallin | Q61597 | 21 | 5 | 2.02 | -2.66 |
|  | γA-crystallin | P04345 | 21 | 2 |  |  |
|  | Ubiquitin-conjugating enzyme E2 | P68037 | 18 | 3 |  |  |
| 6368 | αA-crystallin | Q569M7 | 20 | 11 | 1.09 | 30.1 |
|  | Grifin | Q9D1U0 | 16 | 3 |  |  |
| 6667 | αA-crystallin | Q569M7 | 20 | 7 | 9.46 | -1.69 |
|  | βB2-crystallin | P62696 | 23 | 7 |  |  |
|  | αB-crystallin | P23927 | 20 | 4 |  |  |
|  | βB3-crystallin | Q9JJU9 | 24 | 2 |  |  |
|  | βA3/A1-crystallin | Q9QXC6 | 25 | 1 |  |  |
| 6815 | αA-crystallin | Q569M7 | 20 | 5 | 3.06 | -2.75 |
|  | Histone H2A type 1- B/E | P04908 | 14 | 2 |  |  |
|  | Profilin-1 | P62962 | 15 | 1 |  |  |
| 6841 | β-globin | A8D4K4 | 16 | 12 | -10.73 | 4.99 |
|  | αA-crystallin | Q569M7 | 20 | 2 |  |  |
|  | Peptidyl-prolyl cis-trans isomerase | P177U2 | 18 | 1 |  |  |
|  | Histone H2A type 1- B/E | P04908 | 14 | 1 |  |  |
|  | Histone H4 | P6280J | 11 | 1 |  |  |
| 6847 | β-globin | A8D4K4 | 16 | 11 | -4.11 | 5.08 |
|  | αA-crystallin | Q569M7 | 20 | 3 |  |  |
| 6853 | β-globin | A8D4K4 | 16 | 10 | -4.94 | 4.55 |
|  | αA-crystallin | Q569M7 | 20 | 2 |  |  |
| 6887 | Hemoglobin subunit α | A8DUK4 | 16 | 6 | -3.66 | 4.41 |
|  | β-globin | A8D4K4 | 16 | 4 |  |  |
|  | αB-crystallin | P23927 | 20 | 3 |  |  |
|  | Histone H4 | P6280J | 11 | 2 |  |  |
|  | Histone H2A type 1- B/E | P04908 | 14 | 2 |  |  |
| 6922 | Hemoglobin subunit α | A8DUK4 | 16 | 5 | -3.31 | 4.25 |
|  | Histone H2A type 1-B/E | P04908 | 14 | 2 |  |  |
|  | β-globin | A8D4K4 | 16 | 1 |  |  |
|  | Histone H4 | P6280J | 11 | 2 |  |  |
| 6928 | Hemoglobin subunit α | A8DUK4 | 16 | 4 | -2.38 | 2.91 |
|  | Histone H2A type 1-B/E | P04908 | 14 | 2 |  |  |
|  | Profilin-1 | P62962 | 15 | 1 |  |  |
|  | β-globin | A8D4K4 | 16 | 1 |  |  |

WT, wild type; homo, homozygous
