# Supplementary material for: In Vivo Substrates of the Lens Molecular Chaperones αA-Crystallin and αB-Crystallin
Source: PLoS One. 2014 Apr 23;9(4):e95507. doi: 10.1371/journal.pone.0095507 (PMC3997384; doi:10.1371/journal.pone.0095507)

**SUPPLEMENTARY FIGURES**

Supplementary Figure S1. **2D-DIGE analysis of proteomic changes in whole lenses of 2-day-old mice with knock-in of the αA-R49C mutation.** Protein spots that were picked for analysis from the 2D gels of WT and αA-R49C heterozygous (A) and WT and αA-R49C homozygous lenses (B-D) shown in Figure 1. Quantitative image analysis and mass spectrometry data for identified proteins from these gels are listed in Table1.


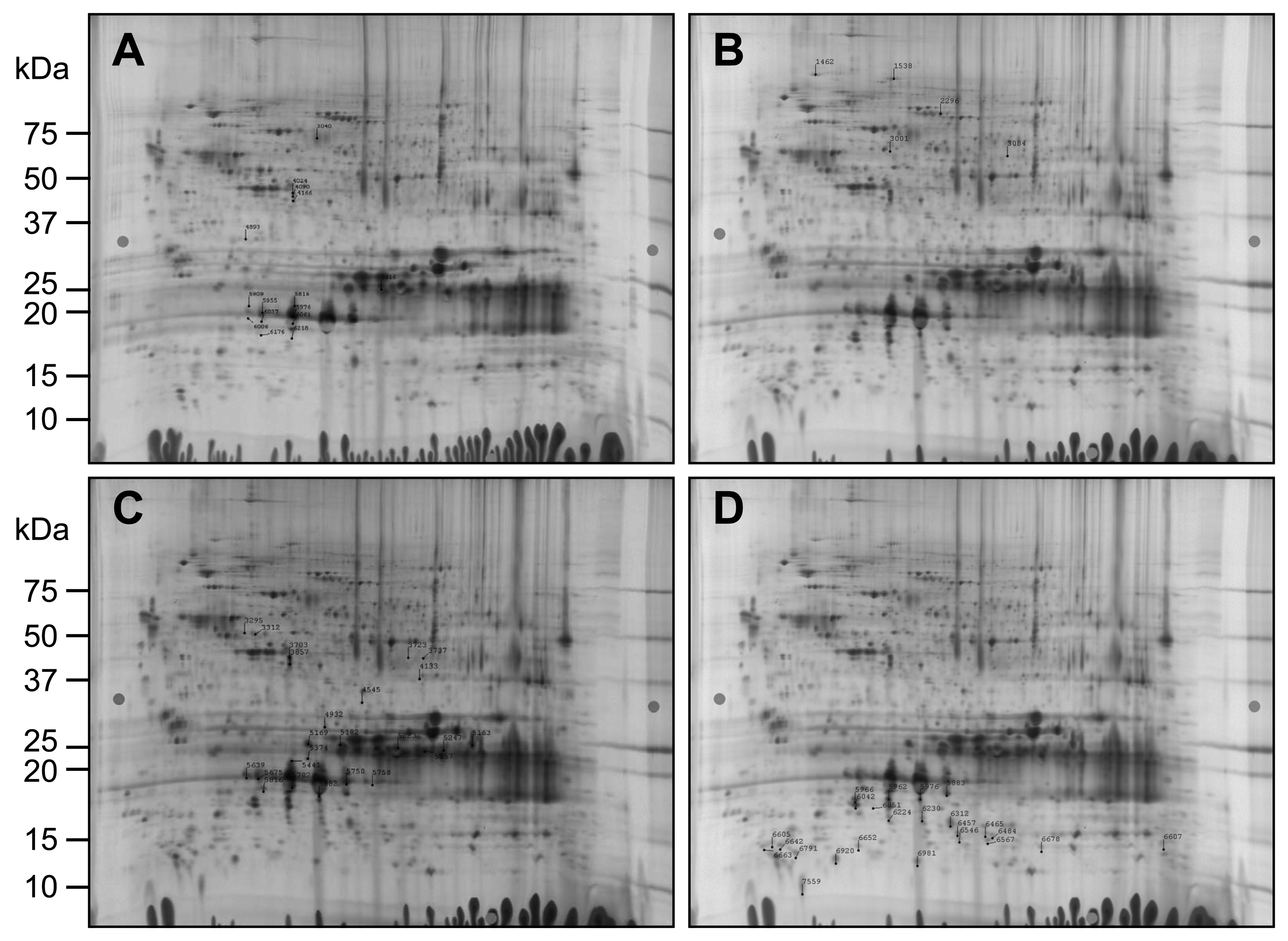


Supplementary Figure S2. **2D-DIGE analysis of proteomic changes in whole lenses of 2-day-old and 14-day-old mice induced by knock-in of the αA-R49C mutation.** (A) A 2D gel of lens proteins labeled with cyanine dyes derived from 2-day-old WT proteins labeled with Cy3, 14-day-old WT proteins labeled with Cy5, and 2-day-old αA-R49C homozygous lens proteins labeled with Cy2. (B, C) Protein spots that were selected for analysis from the gel shown in (A). Proteins were identified by tandem mass spectrometry and Mascot searches of spots that were selected from the gels. Quantitative image analysis and mass spectrometry data for the identified proteins from these gels are listed in Supplemental Table S1.


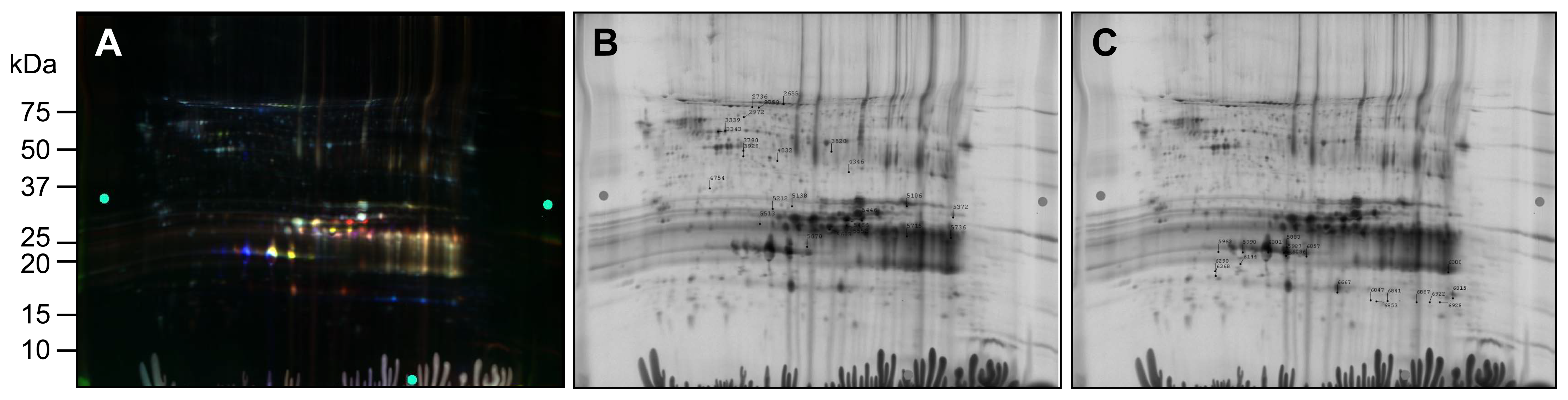


Supplementary Figure S3. **Protein connectivity networks identified by Ingenuity Pathway analysis of lens proteins in αA-R49C knock-in mutant lenses.** Analysis of altered protein networks by Ingenuity Pathway software. Biological networks and pathways generated from input data (Wild type vs. αA-R49C, Tables 1-3 and S1) indicate proteins with changed abundance in gray. (A) A network with GAPDH at the hub. (B) A second network with F-actin at the hub. (C) A third network highlights NPM1 at the hub of the protein connectivity map. (D) A fourth network with TGFB1 at the hub. (E) A fifth network indicates the interaction between grifin and IKZF1. (F) A sixth network shows Gm5409 at the hub. Note that two additional networks are shown in Figure 8.


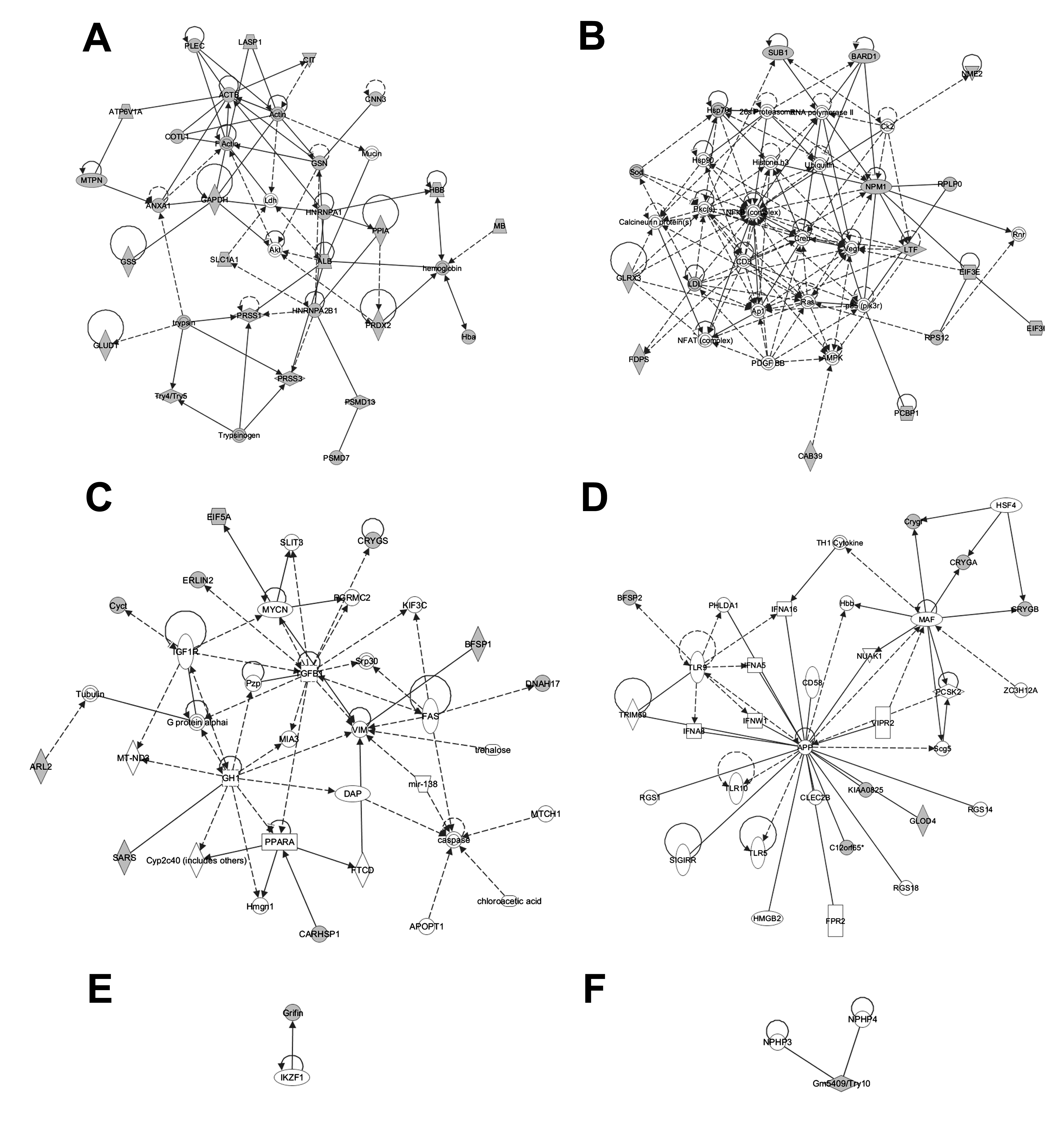


Supplementary Figure S4. **Networks revealed by Ingenuity Pathway analysis of lens proteins that changed in amount in WT vs. αB-R120G knock-in lenses.** Biological networks and pathways generated from input data (Wild type vs. αB-R120G, Table 4) indicate proteins with changed abundance in gray. (A) A network with MAF at the hub. (B) A second network with UBC at the hub. (C) A third network shows the interactions between grifin and IKZF1. (D) A fourth network highlights CTRB2 at the hub of the protein connectivity map.


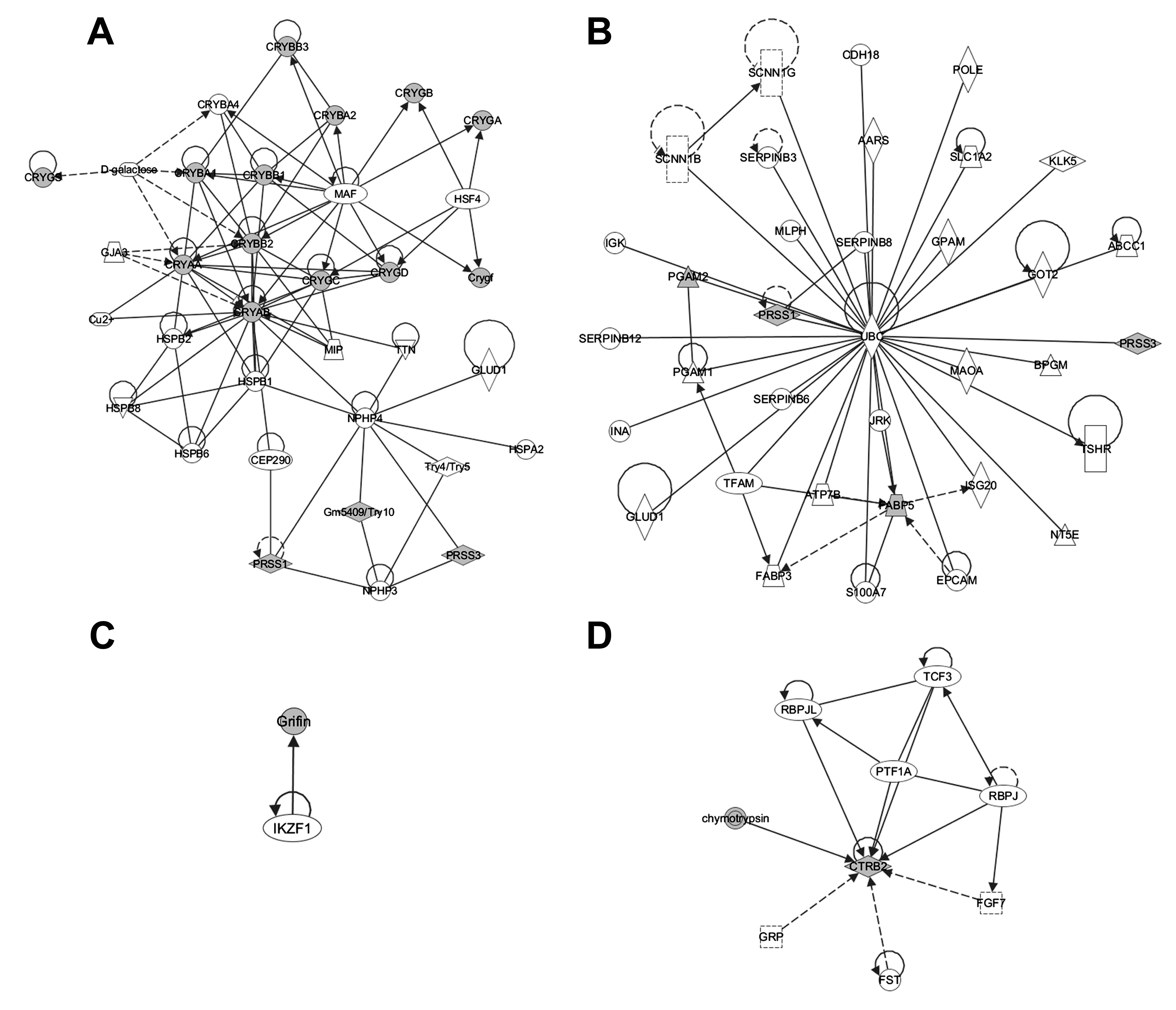

Supplement: File S1 — Supplementary figures. Figure S1, 2D-DIGE analysis of proteomic changes in whole lenses of 2-day-old mice with knock-in of the αA-R49C mutation. Protein spots that were picked for analysis from the 2D gels of WT and αA-R49C heterozygous (A) and WT and αA-R49C homozygous lenses (B-D) shown in Figure 1. Quantitative image analysis and mass spectrometry data for identified proteins from these gels are listed in Table 1. Figure S2, 2D-DIGE analysis of proteomic changes in whole lenses of 2-day-old and 14-day-old mice induced by knock-in of the αA-R49C mutation. (A) A 2D gel of lens proteins labeled with cyanine dyes derived from 2-day-old WT proteins labeled with Cy3, 14-day-old WT proteins labeled with Cy5, and αA-R49C homozygous lens proteins labeled with Cy2. (B, C) Protein spots that were selected for analysis from the gel shown in (A). Proteins were identified by tandem mass spectrometry and Mascot searches of spots that were selected from the gels. Quantitative image analysis and mass spectrometry data for the identified proteins from these gels are listed in Table S1. Figure S3, Protein connectivity networks identified by Ingenuity Pathway analysis of lens proteins in αA-R49C knock-in mutant lenses. Analysis of altered protein networks by Ingenuity Pathway software. Biological networks and pathways generated from input data (Wild type vs. αA-R49C, Tables 1–3 and S1) indicate proteins with changed abundance in gray. (A) A network with GAPDH at the hub. (B) A second network with F-actin at the hub. (C) A third network highlights NPM1 at the hub of the protein connectivity map. (D) A fourth network with TGFB1 at the hub. (E) A fifth network indicates the interaction between grifin and IKZF1. (F) A sixth network shows Gm5409 at the hub. Note that two additional networks are shown in Figure 8. Figure S4, Networks revealed by Ingenuity Pathway analysis of lens proteins that changed in amount in WT vs. αB-R120G knock-in lenses. Biological networks and pathways generated [file pone.0095507.s005.doc]
