# Supplementary material for: Correction: In Vivo Substrates of the Lens Molecular Chaperones αA-Crystallin and αB-Crystallin
Source: PLoS One. 2014 Jul 1;9(7):e102210. doi: 10.1371/journal.pone.0102210 (PMC4077816; doi:10.1371/journal.pone.0102210)
Supplement: File S2 — Republished, corrected article. (PDF) [file pone.0102210.s002.pdf]

OPEN ACCESS Freely available online

PLOS | ONE

In Vivo Substrates of the Lens Molecular Chaperones αA-Crystallin and αB-Crystallin

Usha P. Andley[1,2]*, James P. Malone[4], R. Reid Townsend[3,4]

1 Department of Ophthalmology and Visual Sciences, Washington University School of Medicine, St. Louis, Missouri, United States of America, **2** Department of Biochemistry and Molecular Biophysics, Washington University School of Medicine, St. Louis, Missouri, United States of America, **3** Department of Cell Biology and Physiology, Washington University School of Medicine, St. Louis, Missouri, United States of America, **4** Department of Medicine, Washington University School of Medicine, St. Louis, Missouri, United States of America

Abstract

αA-crystallin and αB-crystallin are members of the small heat shock protein family and function as molecular chaperones and major lens structural proteins. Although numerous studies have examined their chaperone-like activities *in vitro*, little is known about the proteins they protect *in vivo*. To elucidate the relationships between chaperone function, substrate binding, and human cataract formation, we used proteomic and mass spectrometric methods to analyze the effect of mutations associated with hereditary human cataract formation on protein abundance in αA-R49C and αB-R120G knock-in mutant lenses. Compared with age-matched wild type lenses, 2-day-old αA-R49C heterozygous lenses demonstrated the following: increased crosslinking (15-fold) and degradation (2.6-fold) of αA-crystallin; increased association between αA-crystallin and filensin, actin, or creatine kinase B; increased acidification of βB1-crystallin; increased levels of grifin; and an association between βA3/A1-crystallin and αA-crystallin. Homozygous αA-R49C mutant lenses exhibited increased associations between αA-crystallin and βB3-, βA4-, βA2-crystallins, and grifin, whereas levels of βB1-crystallin, gelsolin, and calpain 3 decreased. The amount of degraded glutamate dehydrogenase, α-enolase, and cytochrome c increased more than 50-fold in homozygous αA-R49C mutant lenses. In αB-R120G mouse lenses, our analyses identified decreased abundance of phosphoglycerate mutase, several β- and γ-crystallins, and degradation of αA- and αB-crystallin early in cataract development. Changes in the abundance of hemoglobin and histones with the loss of normal α-crystallin chaperone function suggest that these proteins also play important roles in the biochemical mechanisms of hereditary cataracts. Together, these studies offer a novel insight into the putative *in vivo* substrates of αA- and αB-crystallin.

Citation: Andley UP, Malone JP, Townsend RR (2014) *In Vivo* Substrates of the Lens Molecular Chaperones αA-Crystallin and αB-Crystallin. PLoS ONE 9(4): e95507. doi:10.1371/journal.pone.0095507

Editor: K. Krishna Sharma, University of Missouri-Columbia, United States of America

Received February 7, 2014; **Accepted** March 26, 2014; **Published** April 23, 2014

Copyright: © 2014 Andley et al. This is an open-access article distributed under the terms of the Creative Commons Attribution License, which permits unrestricted use, distribution, and reproduction in any medium, provided the original author and source are credited.

Funding: This work is supported by the National Institutes of Health (NIH) Grant R01EY05681-29 to U.P.A.; Core Grant EY02687; Research to Prevent Blindness grants to the Department of Ophthalmology and Visual Sciences at Washington University School of Medicine, Washington University Institute of Clinical and Translational Sciences; Grant UL1 TR000448 from the National Center for Advancing Translational Sciences (NCATS) of the NIH; grants from the National Center for Research Resources (5P41RR000954-35); and a grant from the National Institute of General Medical Sciences (8 P41 GM103422-35). The content is solely the responsibility of the authors and does not necessarily represent the official view of the NIH. The funders had no role in study design, data collection and analysis, decision to publish, or preparation of the manuscript.

Competing Interests: Usha Andley is an Editorial Board member for PLOS ONE. This does not alter the authors' adherence to PLOS ONE Editorial Policies and criteria.

* Email: andley@vision.wustl.edu

Introduction

α-crystallins are major proteins of lens fiber cells that comprise approximately 35% of the water-soluble lens protein and are essential for lens transparency. Mutations in α-crystallin genes are known to cause hereditary cataracts in humans. However, the cellular functions of α-crystallin in maintaining growth, development, and transparency of the lens and the mechanisms by which loss of α-crystallin function leads to cataracts are not fully understood.

The vertebrate lens expresses two α-crystallin proteins, αA and αB, at a high concentration in lens fiber cells and at lower levels in the lens epithelium [1–4]. Transcription of αA and αB-crystallin genes commences early in lens development, beginning at embryonic day 10.5 and 9.5 respectively in the mouse, and continues as the lens matures [5]. In lens fiber cells, α-crystallins form heteroaggregates of αA- and αB-crystallins in a 3:1 ratio [6]. αA- and αB-crystallins are members of the small heat shock protein family of molecular chaperones [7]. Homo-oligomers of αA-crystallin and αB-crystallin and the α-crystallin heteroaggregates possess chaperone-like activity, binding to partially unfolded or denatured proteins to suppress non-specific aggregation [7].

The molecular mechanisms by which point mutations in crystallin genes lead to hereditary human cataract formation are not completely understood [8–11]. Mouse models carrying naturally occurring α-crystallin mutations have provided valuable information on the functions of these mutant proteins *in vivo* [12–14].

The R49C mutation in αA-crystallin was found to be associated with nuclear cataract in four generations of a Caucasian family [15]. The mutant protein is mislocalized to the nucleus, and has reduced solubility [15,16]. Most notably, this mutation is in the N-terminal region of αA-crystallin, a region thought to be important for aggregation interactions [16]. In mice, the R49C mutant produces a small eye/lens phenotype and severe cataracts at birth in 100% of mice homozygous for the mutation, indicating a gain

in toxic function of αA-crystallin protein. Compared with homozygous mice, heterozygous αA-R49C knock-in mice, which mimic human cataract patients, develop cataracts at approximately 2 months of age and exhibit decreased protein solubility and altered cell signaling. Moreover, the R49C mutation significantly alters interactions between αA-crystallin, αB-crystallin, βB2-crystallin, γ-crystallins, and the cytoskeletal protein tubulin. The αB-R120G mutation in αB-crystallin also causes cataracts in humans [8]. αB-R120G knock-in mice have lens opacities, which are evident even in 3-week-old animals [17]. We found that 100% of heterozygous mice ranging in age from 3 weeks to 5 months had lens opacities, with severity increasing with age. Homozygous mice also developed lens opacities, but the effect did not appear to be dependent on mutant gene dosage.

Our novel studies using knock-in mouse models for these mutations have shown profound effects on the lens and eye and indicate that α-crystallins affect lens epithelial and fiber cell growth and survival, in addition to their well-known role in transparency and optical properties of the lens. Moreover, our data suggest that αA- and αB-crystallin mutations alter the structure and function of lens epithelial and fiber cells and exert toxic effects at an early stage of development, when primary fiber cell differentiation commences.

It is well established that abnormal interactions between chaperone and substrate proteins can result in increased protein aggregation and disease [8,18]. The substrate-chaperone interaction between αB-crystallin and its substrates involves multiple interactive domains that have been extensively characterized [19,20]. However, the *in vivo* substrates of αA- and αB-crystallin in the lens have not been identified. In the absence or reduction of α-crystallin chaperone function, it is likely that partially unfolded proteins will accumulate and aggregate [21,22]. We therefore focused on determining which proteins are associated with α-crystallin chaperones with the aim of identifying proteins that are dependent on the chaperone activity of αA- and αB-crystallins to retain their native conformations *in vivo*. To achieve this, we analyzed the abundance of proteins in αA-R49C and αB-R120G knock-in mutant mice lenses by proteomics and mass spectrometry. We have already applied this approach to identify several proteins and enzymes not previously known to be affected by αA- or αB-crystallin loss of function [23]. This method has also been used to identify the effect of loss of function of the heat shock chaperone protein HSP90 [24].

Results

Two-day-old αA-R49C Mouse Lenses

To identify proteins that showed altered abundance in mouse lenses with the R49C αA-crystallin mutation, we performed 2D-DIGE of 2-day-old WT, αA-R49C heterozygous mutant, and αA-R49C homozygous mutant lenses. Figure 1 and Fig. S1 in File S1 show 2D gels of proteins and Table 1 lists the approximately 100 protein spots that showed a change in abundance between these samples. Figure 2 shows the 3D plots for some of the spots that changed in abundance in these lenses. Compared with WT, αA-R49C heterozygous lenses had a 15-fold higher abundance of crosslinked αA-crystallin, a 3-fold higher abundance of more acidic αA-crystallin, and a 2.6-fold higher abundance of degraded αA-crystallin. The association of αA-crystallin with filensin increased 17-fold, the association of αA-crystallin with actin and creatine kinase B increased 15-fold, and the amount of actin alone increased 10.79-fold. The amount of a more acidic form of βB1-crystallin increased, whereas that of a basic form of βB1-crystallin decreased. αA-crystallin associated with βA3/A1 was more acidic and had a slightly lower apparent molecular weight than free αA-crystallin. The number of protein spots with altered abundance was much greater in the αA-R49C homozygous mutant lenses than in the heterozygous lenses. In the homozygous lenses, several proteins in the high molecular weight region (>75 kDa) were altered. A high-molecular weight crosslinked αA-crystallin associated with creatine kinase B, actin, and erlin was enhanced 15-fold. The association of αA-crystallin with α-enolase and βA3/A1 was also enhanced in homozygous lenses. In the same lenses, the amount of βB1-crystallin decreased and more acidic forms of βB1- and βB3-crystallins were associated with αA-crystallin. Among proteins in the 20-kDa region (Table 1, Fig. 1 and Fig. S1 in File S1), the amount of αA-crystallin and βA3/A1-crystallin decreased in homozygous lenses. Among the cytoskeletal proteins, the levels of more basic forms of filensin and phakinin decreased, whereas levels of more acidic forms of these proteins increased. High molecular weight forms of phakinin and actin decreased 2.9-fold in homozygous lenses. The amount of tubulin, vimentin, and microtubule associated protein RP/EB associated with αA-crystallin increased in homozygous lenses, while that of phosphoglycerate mutase decreased. The amount of hemoglobin subunit 1 complexed with γD-, αB-, γS-, γB-, βB3-, and γA-crystallins decreased in homozygous lenses and increased with age. The abundance of forms of Hsp71 increased 2.5-fold, and the amount of αA-crystallin associated with vimentin, tubulin, and T-complex protein increased 4-fold in homozygous lenses. The amount of grifin associated with αA-crystallin increased in several spots.

There was an increase in β-globin, histone and peptidyl-prolyl cis-trans isomerase associated with αA-crystallin in homozygous lenses (Table S1). The abundance of αB-crystallin, hemoglobin, and histones also increased. A spot containing a high molecular weight form of spectrin-α and nucleosome assembly protein increased in homozygous lenses. In the high molecular weight region, the abundance of αA-crystallin and spectrin increased and that of filensin, gelsolin, and calpain 3 decreased in homozygous lenses. There was an increase in mitochondrial 60-kDa HSP associated with αA-crystallin, and many other proteins including vimentin.

Among proteins in the cytoskeletal and 20 kDa regions (Table 1, Fig. 1 and Fig. S1 in File S1), there was an increase in αA-crystallin associated with βB3-crystallin, βA4-crystallin, grifin, fatty acid binding protein, thymosin, and glutamate dehydrogenase in homozygous lenses. Surprisingly, the amount of αA-crystallin alone and in association with βA3/A1-crystallin, βA4-crystallin, γE-crystallin, and γA-crystallin in the high molecular weight region decreased in homozygous lenses.

Increased amounts of degraded proteins were detected in the low molecular weight region (<20 kDa). The amount of degraded glutamate dehydrogenase alone and in association with cytochrome c increased 4-fold and 53-fold, respectively, in homozygous lenses. The amount of more acidic forms of αA-crystallin, and more degraded forms of creatine kinase B, αA-crystallin, actin, and phakinin increased 19-fold in homozygous lenses. In the molecular weight range below 20 kDa, the amount of degraded αB-crystallin associating with βA2-crystallin, α-enolase, and other proteins increased 112-fold in homozygous lenses. The amount of other degradation products of αA-crystallin associated with β- and γ-crystallins also increased in homozygous lenses. Some of these were more basic than the original αA-crystallin. The amount of a very acidic cohort of αA-crystallin with βA3/A1-crystallin, hemoglobin subunit α, and G3PDH increased 7-fold in homozygous lenses. There was also an increase in the amount of a very low molecular weight αA-crystallin associated with stathmin and other β-crystallins in homozygous lenses.

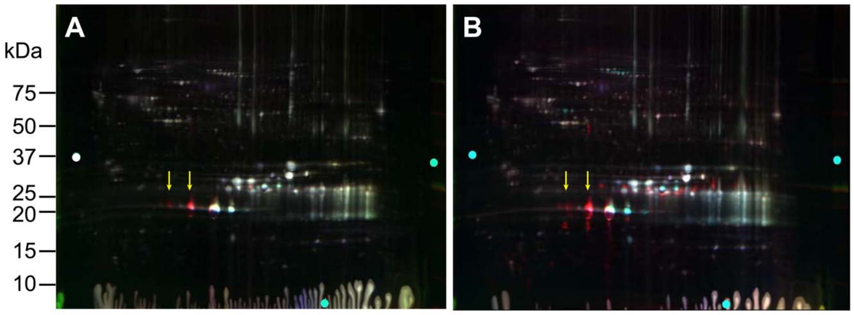

Figure 1. 2D-DIGE analysis of proteomic changes in whole lenses of 2-day-old mice with knock-in of the αA-R49C mutation. (A) 2D gel of cyanine dye-labeled lens proteins derived from wild-type sample 1 (WT1) proteins labeled with Cy2, WT2 proteins labeled with Cy3, and αA-R49C heterozygous proteins labeled with Cy5. (B) 2D gel of cyanine dye-labeled lens proteins derived from WT1 proteins labeled with Cy2, WT2 proteins labeled with Cy3, and αA-R49C homozygous proteins labeled with Cy5. Protein spots that were selected for analysis from the gels shown in (A) and (B) are shown in Fig. S1 in File S1 and were identified by tandem mass spectrometry and Mascot searches. Quantitative image analysis and mass spectrometry data for the identified proteins are listed in Table 1. Arrows indicate the shift in position of the αA-crystallin bands *(red)* to a more acidic pI with the mutation.
doi:10.1371/journal.pone.0095507.g001

Previous work demonstrated that there is less insoluble protein in heterozygous lenses than in homozygous lenses [10]. To determine whether changes in protein abundance reflect this difference in solubility, equal amounts of WT, heterozygous, and homozygous mutant lens proteins were further analyzed on multiple gels using various combinations of cyanine dyes to label WT and mutant lens samples. Multi-gel analysis of WT and αA-R49C mutant proteins is shown in Table 2 and Figures 3 and 4. Biological variation analysis (BVA) of WT and αA-R49C heterozygous and homozygous lenses showed that mutant gene dosage correlated with an increase in alanyl-tRNA synthetase, αA-crystallin, the mammalian cytoplasmic chaperone TCP-1 theta, and high-molecular weight βA3/A1-crystallin. The statistical significance of the change in protein abundance of each spot is shown in Table 2. The levels of two different members of the HSP70 protein family, HSC70 and mitochondrial stress protein 70, as well as the V-type proton ATPase catalytic subunit, also increased in αA-R49C mutant lenses. Mitochondrial stress protein 70 increased in two spots (spots 928 and 948) and TCP-1 associated with αA-crystallin increased in three spots (spots 593, 1081, and 1146). High molecular weight βB1-crystallin increased slightly in a mutation- and dose-dependent manner. The abundance of βA3/A1-crystallin associated with αA-crystallin (spot 1477) and αA-crystallin alone (spot 1612) decreased. It is noteworthy that for several spots, the differences were statistically significant ($p<0.05$) between WT and the αA-R49C homozygous lenses only. The 79-fold increase in αA-crystallin (spot 1540) in the high molecular weight region was highly significant, suggesting increased crosslinking of αA-crystallin in αA-R49C mutant lenses. Creatine kinase B associated with αA-crystallin in the high molecular weight region increased 22-fold (spot 1519), confirming the results of the single gel analysis in Table 1. The amount of αA-crystallin associated with eukaryotic translational initiation factor increased 1.44- and 2.24-fold in heterozygous and homozygous mutant lenses, respectively. Among the proteins that showed decreased abundance in a mutation- and dosage-dependent manner were βB1-crystallin (spots 1856 and 1868) associated with eukaryotic translational initiation factor, αA-crystallin associated with histone H4, implantin, myotrophin, and more basic αA-crystallin associated with βA4- and βA3/A1-crystallins in spot 2772.

Additional proteins that decreased in abundance relative to wild type (Fig. 4 and Table 2) were βB1-crystallin (in homozygous lenses only), and a mutation- and dose-dependent decrease in βA3/A1-, βA4-, βA2-crystallins associated with αA-crystallin (spot 2109), αB-crystallin, and βB2-crystallin (spots 2115 and 2123 showed a 8.57-fold decrease in homozygous lenses relative to WT). The abundance of γD-crystallin, peptidyl-prolyl cis-trans isomerase, γA-crystallin, γB-crystallin, and γC-crystallin also decreased (spot 2413). Other spots that decreased in abundance in a mutation- and dose-dependent manner were nucleoside diphosphate kinase, peptidyl-prolyl cis-trans isomerase, and γD-crystallin (spot 2454), fatty acid-binding protein and αA-crystallin (spot 2553). A more acidic form of αA-crystallin increased 4- and 5-fold in heterozygous and homozygous lenses (spot 2294). In contrast, spot 2317 decreased 4.8- and 9.2-fold in heterozygous and homozygous mutant lenses, respectively. Spot 2351 increased in a mutation- and dose-dependent manner with 4.6- and 10.4-fold increases in heterozygous and homozygous lenses, respectively. Spots 2317 and 2351 contained only αA-crystallin at its normal molecular weight, but spot 2351 was more acidic, suggesting a decrease in the pI of αA-crystallin by the R49C mutation. Spot 2417, containing only a lower-than normal molecular weight αA-crystallin also increased 7.5- and 10.5-fold in αA-R49C mutant lenses relative to WT, but two additional spots containing only αA-crystallin decreased (spots 2533 and 2631). The abundance of epidermal fatty acid binding protein and 40S ribosomal protein S12 also decreased in association with αA-crystallin, but these changes were not mutation- and dose-dependent.

Two-week Old αA-R49C Mouse Lenses

Figure 5 shows 2D gels for 14-day-old WT and mutant proteins of αA-R49C knock-in mice. Table 3 shows the approximately 50 protein spots that showed a change in abundance between WT and αA-R49C mutant in 14-day-old lenses. The abundance of the high molecular weight cytoskeletal protein spectrin-α and its acidic forms decreased in αA-R49C lenses (spots 700 and 769). Acidic forms of filensin increased 4-fold (spot 2675), whereas basic forms decreased 15-fold (spot 2448). Hsp70 also increased 3- to 6-fold in three spots. High molecular weight phakinin decreased 10-fold, while acidic and low molecular weight phakinin increased 8-fold.

Among the crystallins, the amount of αA-crystallin that was crosslinked and associated with βA3/A1-crystallin increased in four spots, and αA-crystallin associated with annexin increased 3-fold in one spot (spot 4872). Normal and basic forms of βB1-crystallin decreased 6- to 25-fold in three spots. More basic forms

Table 1. Single-gel analysis of proteins that showed differences in abundance between 2-day-old WT and heterozygous or homozygous αA-R49C lenses.

Spot number	Protein	UNIPROT accession number	MW (kDa)	Number of assigned spectra	Fold change		
					WT1 vs. WT2	WT1 vs. heterozygous	WT2 vs. heterozygous
3040	serum albumin	P07724	69	39	3.67	17.57	16.1
	αA-crystallin	Q569M7	20	5			
	Filensin	A2AMT1	74	3			
4024	αA-crystallin	Q569M7	20	11	1.48	16.89	14.83
	Actin cytoplasmic	P62737	42	5			
	Creatine kinase B	Q04447	43	4			
	Erlin-2	Q8BFZ9	38	3			
4090	αA-crystallin	Q569M7	20	16	1.45	15.56	11.46
	Actin cytoplasmic	P62737	42	5			
	Creatine kinase B	Q04447	43	3			
	βA3/A1-crystallin	Q9QXC6	25	1			
4166	αA-crystallin	Q569M7	20	1 (99%)	1.39	15.09	10.67
4893	Actin cytoplasmic	P62737	42	1	1.39	10.79	8.77
	Citron Rho-interacting kinase	P49025	235	1			
	14-3-3 protein sigma	O70456	28	1			
	Peroxiredoxin-2	Q61171	22	1			
	Glyoxalase domain-containing protein 4	Q9CPV4	20	1			
5616	αA-crystallin	Q569M7	20	21	1.24	7.51	7.46
	βA3/A1-crystallin	Q9QXC6	25	17			
	βA2-crystallin	Q9QXC6	22	3			
	βA4-crystallin	Q3TSJ3	24	2			
5816	αA-crystallin	Q569M7	20	19	1.22	6.2	6.91
	βA3/A1-crystallin	Q9QXC6	25	5			
	γA-crystallin	Q6PGI0	21	3			
	γD-crystallin	P04345	21	2			
5909	αA-crystallin	Q569M7	20	11	1.17	6	6.83
	βA3/A1-crystallin	Q9QXC6	25	3			
	γA-crystallin	P04345	21	2			
	γD-crystallin	Q6PGI0	21	2			
	γB-crystallin	P04344	21	2			
	Eukaryotic trans initiation factor	P63242	17	1			
5955	αA-crystallin	Q569M7	20	12	1.12	5.41	4.76
	γA-crystallin	P04345	21	2			

Table 1. Cont.

Spot number	Protein	UNIPROT accession number	MW (kDa)	Number of assigned spectra	Fold change		
					**WT1 vs. WT2**	**WT1 vs. heterozygous**	**WT2 vs. heterozygous**
	βA3/A1-crystallin	Q9QXC6	25	2			
	γD-crystallin	Q6PGI0	21	1			
5976	αA-crystallin	Q569M7	20	17	1.08	4.89	4.57
	βA3/A1-crystallin	Q9QXC6	25	2			
	γA-crystallin	P04345	21	2			
6006	αA-crystallin	Q569M7	20	14	1.08	3.17	3.68
	Eukaryotic trans initiation factor	P63242	17	1			
6037	αA-crystallin	Q569M7	20	19	−1.11	2.97	3.63
	βA3/A1-crystallin	Q9QXC6	25	2			
6061	αA-crystallin	Q569M7	20	37	−1.13	2.62	2.5
	βA3/A1-crystallin	Q9QXC6	25	1			
	γA-crystallin	P04345	21	1			
6176	αA-crystallin	Q569M7	20	14	−1.21	1.86	2.15
	Activated RNA polymerase II transcriptional coactivator p15	P11031	14	4			
6218	αA-crystallin	Q569M7	20	14	−1.75	1.74	−2.06
	Grifin	Q9D1U0	16	3			
	βA3/A1-crystallin	Q9QXC6	25	2			
Spot number	Protein	UNIPROT accession number	MW (kDa)	Number of assigned spectra	Fold change		
					WT1 vs. WT2	WT1 vs. homozygous	WT2 vs. homozygous
1462	Spectrin-α	A3KGU5	283	16	1.09	10.15	9.34
	Nucleosome assembly protein 1-like 4	Q792Z1	43	1			
1538	Spectrin-α	A3KG45	283	59	1.21	8.33	6.91
	Neuronal cell adhesion molecule	Q810U4	139	8			
	αA-crystallin	Q569M7	20	5			
	Serrate RNA effector molecule homolog	Q99MR6	100	2			
	Methionine synthase	A6H5Y3	139	2			
2296	Filensin	A2AMT1	74	43	−1.07	−5.61	−5.19
	Gelsolin	P13020	86	5			
	Calpain 3	A2AVV5	85	4			
3001	60 kDa Heat shock protein, mitochondrial	P63038	61	16	−1.11	6.23	6.95
	T-complex protein 1 subunit theta	P42932	60	9			
	αA-crystallin	Q569M7	20	5			

Table 1. Cont.

Spot number	Protein	UNIPROT accession number	MW (kDa)	Number of assigned spectra	Fold change		
					WT1 vs. WT2	WT1 vs. heterozygous	WT2 vs. heterozygous
	Tubulin alpha-1A chain	P68369	50	4			
	Tubulin beta-5 chain	P99024	50	2			
	Vimentin	P20152	54	1			
	Glutathione synthetase	P51855	52	1			
3084	Ezrin	P26040	69	10	1.28	3.98	3.12
	Fascin	Q61553	55	5			
	αA-crystallin	Q569M7	20	5			
	D-3-phosphoglycerate dehydrogenase	Q61753	57	3			
	α-Enolase	P17182	47	2			
	βB1-crystallin	Q9WVJ5	28	2			
	Aspartyl-tRNA synthetase, cytoplasmic	Q922B2	57	1			
3295	αA-crystallin	Q569M7	20	15	1.01	−2.92	−2.92
	βA3/A1-crystallin	Q9QXC6	25	5			
	βA4-crystallin	Q9JJV0	22	4			
	γE-crystallin	QO3740	21	3			
	γB-crystallin	PO4344	21	2			
	Superoxide dismutase [Cu-Zn]	P08228	16	2			
3312	αA-crystallin	Q569M7	20	23	−1.12	−5.51	−4.9
3703	αA-crystallin	Q569M7	20	13	1.01	14.68	14.6
	Filaggrin-2	Q5D862	248	1			
3723	αA-crystallin	Q569M7	20	14	−1.58	2.99	4.75
	βA3/A1-crystallin	Q9QXC6	25	3			
	βA4-crystallin	Q9JJV0	22	3			
	βB3-crystallin	Q9JJU9	24	1			
	Superoxide dismutase [Cu-Zn]	P08228	16	1			
3737	αA-crystallin	Q569M7	20	14	1.09	4.9	4.53
3857	αA-crystallin	Q569M7	20	15	1.09	31.67	29.19
	Grifin	Q9D1U0	16	1			
4133	αA-crystallin	Q569M7	20	11	1.22	3.93	3.24
	Grifin	Q9D1U0	16	2			
4545	αA-crystallin	Q569M7	20	11	1.13	6.68	5.93
	βA4-crystallin	Q9JJV0	22	2			
	βA3/A1-crystallin	Q9QXC6	25	2			

Table 1. Cont.

Spot number	Protein	UNIPROT accession number	MW (kDa)	Number of assigned spectra	Fold change		
					WT1 vs. WT2	WT1 vs. heterozygous	WT2 vs. heterozygous
4932	Fatty acid binding protein epidermal	Q05816	20	18	−1.01	2.97	3.01
	αA-crystallin	Q569M7	20	8			
	βA4-crystallin	Q9JJV0	22	2			
5163	αA-crystallin	Q569M7	20	5	1.00	4.26	4.28
	Fatty acid binding protein epidermal	Q05816	20	3			
5169	βB3-crystallin	Q9JJU9	24	6	1.33	21.6	16.28
	αA-crystallin	Q569M7	20	4			
	βA2-crystallin	Q9JJV1	22	1			
5182	αA-crystallin	Q569M7	20	5	−1.41	6.57	9.3
	βB3-crystallin	Q9JJU9	24	4			
	Calpain-3	Q64691	94	1			
	βA3/A1-crystallin	Q9QCX6	25	1			
5223	αA-crystallin	Q569M7	20	6	−1.06	−4.42	−4.15
5247	αA-crystallin	Q569M7	20	4	−1.05	2.69	2.84
	βB3-crystallin	Q9JJU9	24	2			
5257	αA-crystallin	Q569M7	20	3	1.05	3.33	3.21
	Thymosin beta-4	P20065	6	1			
5441	αA-crystallin	Q569M7	20	4	1.43	8.35	5.85
5639	αA-crystallin	Q569M7	20	5	−1.05	4.83	5.1
5675	αA-crystallin	Q569M7	20	5	1.24	8.29	6.71
	Thymosin beta-4	P20065	6	2			
5816	αA-crystallin	Q569M7	20	7	−1.21	6.45	7.83
	βA3/A1-crystallin	Q9QCX6	25	2			
	βA4-crystallin	Q9KKV0	22	2			
5883	Glutamate dehydrogenase	P26443	61	3	1.24	4.01	3.26
5966	Creatine kinase B-type	Q04447	43	8	−1.1	19.82	22.03
	αA-crystallin	Q569M7	20	7			
	Actin cytoplasmic 1	P60709	42	5			
	COP9 signalosome complex subunit 4	O88544	46	4			
	26 S proteasome non-ATPase regulatory subunit 13	Q9WJJ2	43	3			
	Erlin-2	Q8BFZ9	38	2			
	Activator of 90 kDa HSP ATPase homolog 1	Q8BK64	38	2			

Table 1. Cont.

Spot number	Protein	UNIPROT accession number	MW (kDa)	Number of assigned spectra	Fold change		
					WT1 vs. WT2	WT1 vs. heterozygous	WT2 vs. heterozygous
	Eukaryotic initiation factor 4A-I	P60843	46	1			
	Succinyl-CoA subunit beta	Q9Z2I9	38	1			
	Farnesyl pyrophosphate synthase	Q920E5	50	1			
	Glutaredoxin-3	Q9CQM9	38	1			
5976	βB3-crystallin	Q9JJU9	24	8	−1.17	14.73	17.35
	αA-crystallin	Q569M7	20	6			
	βA2-crystallin	Q9JJV1	22	3			
	βB2-crystallin	P62696	23	2			
	Gelsolin	P13020	86	2			
	βA3/A1-crystallin	Q9QXC6	25	1			
	γN-crystallin	Q8VHL5	21	1			
6042	αA-crystallin	Q569M7	20	5	1.13	112.49	100.21
	βB3-crystallin	Q9JJU9	24	5			
	α-Enolase	P17182	47	3			
	βA2-crystallin	Q9JJV1	22	2			
	βB1-crystallin	Q9WVJ5	28	2			
	Poly(γC)-binding protein 1	P60335	37	2			
	βA3/A1-crystallin	Q9QXC6	25	1			
	γN-crystallin	Q8VHL5	21	1			
6051	Glutamate dehydrogenase mitochondrial	P26443	61	4	−1.27	3.73	4.75
	Myoglobin	P04249	17	1			
6224	αA-crystallin	Q569M7	20	5	1.34	23.76	17.83
	βB3-crystallin	Q9JJU9	24	4			
	βA2-crystallin	Q9JJV1	22	2			
	βA3/A1-crystallin	Q9QXC6	25	1			
6312	αA-crystallin	Q569M7	20	7	1.13	−2.72	−3.04
	βB3-crystallin	Q9JJU9	24	2			
	βA3/A1-crystallin	Q9QXC6	25	2			
6457	βB3-crystallin	Q9JJU9	24	18	1.19	5.94	5.03
	αA-crystallin	Q569M7	20	5			
	γB-crystallin	Q6PHP7	21	3			
	γF-crystallin	Q9CXV3	21	3			
	Triosephosphate isomerase	P17751	27	1			

Table 1. Cont.

Spot number	Protein	UNIPROT accession number	MW (kDa)	Number of assigned spectra	Fold change		
					WT1 vs. WT2	WT1 vs. heterozygous	WT2 vs. heterozygous
	αB-crystallin	P23927	20	1			
6465	βB1-crystallin	Q9WVJ5	28	18	−1.01	3.29	3.35
	βA3/A1-crystallin	Q9QXC6	25	5			
	Proteasome subunit 1 type 4	P99026	29	5			
	αA-crystallin	Q569M7	20	4			
	βB3-crystallin	Q9JJU9	24	2			
	βA4-crystallin	Q9JJV0	22	2			
	βA2-crystalin	Q9JJV1	22	1			
6484	αA-crystallin	Q569M7	20	6	1.34	6.74	5.05
	βB1-crystallin	Q9WVJ5	28	5			
	βA3/A1-crystallin	Q9QXC6	25	3			
	Proteasome subunit 1 type 4	P99026	29	2			
	βB3-crystallin	Q9JJU9	24	1			
6546	αA-crystallin	Q569M7	20	10	1.78	5.92	3.34
	βB3-crystallin	Q9JJU9	24	9			
	βB1-crystallin	Q9WVJ5	28	5			
	βA3/A1-crystallin	Q9QXC6	25	4			
	βA2-crystallin	Q9JJV1	22	3			
	βS-crystallin	O35486	21	1			
6567	αB-crystallin	P23927	20	17	1.54	9.13	5.96
	βB3-crystallin	Q9JJU9	24	8			
	αA-crystallin	Q569M7	20	5			
	βS-crystallin	O35486	21	5			
	γF-crystallin	Q9CXV3	21	4			
	βA3/A1-crystallin	Q9QXC6	25	3			
	βA2-crystallin	Q9JJV1	22	1			
6605	βA3/A1-crystallin	Q9QXC6	25	15	−1.32	4.39	5.83
	βB3-crystallin	Q9JJU9	24	12			
	αB-crystallin	P23927	20	7			
	αA-crystallin	Q569M7	20	5			
	βA2-crystallin	Q9JJV1	22	5			
	βS-crystallin	O35486	21	3			
	γF-crystallin	Q9CXV3	21	2			

Table 1. Cont.

Spot number	Protein	UNIPROT accession number	MW (kDa)	Number of assigned spectra	Fold change		
					WT1 vs. WT2	WT1 vs. heterozygous	WT2 vs. heterozygous
6607	αA-crystallin	Q569M7	20	12	−1.04	2.82	2.95
	γB-crystallin	Q6PHP7	21	6			
	αB-crystallin	P23927	20	4			
	βB1-crystallin	Q9WVJ5	28	4			
	Calcium-regulated heat stable protein 1	Q9CR86	16	3			
	γF-crystallin	Q9CXV3	21	2			
	βA3/A1-crystallin	Q9QXC6	25	2			
	βA2-crystallin	Q9JJV1	22	1			
	βA4-crystallin	Q9JJV0	22	1			
	γC-crystalin	Q61597	21	1			
6642	αA-crystallin	Q569M7	20	5	−1.18	10.22	12.14
	βA3/A1-crystallin	Q9QXC6	25	1			
	γB-crystallin	Q6PHP7	21	1			
6652	αA-crystallin	Q569M7	20	7	1.17	9.1	7.79
6663	αA-crystallin	Q569M7	20	6	−1.21	6.79	8.29
6678	Hemoglobin subunit α	P01942	15	1	1.34	11.01	8.26
	Glyceraldehyde-3-phosphate	P25856	42	1			
	Hemoglobin subunit β	P11758	16	1			
6791	αA-crystallin	Q569M7	20	5	1.27	10.07	7.96
	βA3/A1-crystallin	Q9QXC6	25	3			
	βB3-crystallin	Q9JJU9	24	2			
6920	αA-crystallin	Q569M7	20	76	−1.23	21.71	26.75
6981	αA-crystallin	Q569M7	20	5	−1.00	26.33	26.6
	γB-crystallin	Q6PHP7	21	1			
	Activated RNA polymerase II transcriptional coactivator P15	P11031	14	1			
	Adenosine receptor A2B	Q60614	36	1			
7559	αA-crystallin	Q569M7	20	15	−2.29	8.38	19.32
	βA3/A1-crystallin	Q9QXC6	25	5			
	Stathmin	P54227	17	4			
	βB3-crystallin	Q9JJU9	24	3			
	βA4-crystallin	Q9JJV0	22	2			
	Superoxide dismutase [Cu-Zn]	P08228	16	1			

Table 1. Cont.

Spot number	Protein	UNIPROT accession number	MW (kDa)	Number of assigned spectra	Fold change		
					WT1 vs. WT2	WT1 vs. heterozygous	WT2 vs. heterozygous
	Fatty acid binding protein, epidermal	Q05816	15	1			

WT, wild-type.
doi:10.1371/journal.pone.0095507.t001

of βB2- and βB3-crystallins in association with glutathione S-transferase-μ (GST-μ) increased 8-21-fold in two spots. Very acidic forms of βB2-crystallin, βB3-crystallin, and GST-μ decreased 18-fold in spot 5625.

αB-crystallin that was degraded and associated with β- and γ-crystallins increased in two spots. The amount of αA-crystallin slightly larger than 20 kDa decreased 60- to 71-fold (spot 6341), and 13-fold when associated with β- and γ-crystallins (spot 6352). Acidic and degraded αA-crystallin increased 34-fold (spot 6485). Spots containing γA-, γB-, γC-, and γD-crystallins decreased 6-fold. Degraded αA-crystallin associated with γC-, γA-, and γB-crystallins increased 15-fold. Nine spots containing degraded αA-crystallin increased in mutant lenses, whereas degraded but more basic forms than the original αA-crystallin decreased in abundance (spots 7068, 7089, and 7419). Wild type and αA-R49C homozygous lenses were further analyzed (Fig. S2 in File S1 and Table S1). There was a large change in βB2-crystallin expression with age of the wild type lenses (from 2 days to 2 weeks). Spots 5446 and 5466 (Table S1) show an increase in βB2-crystallin in wild type mouse lenses confirming the results of a previous study [23].

Two-week Old αB-R120G Mouse Lenses

Figure 6 shows 2D gels for 14-day-old WT and mutant proteins of αB-R120G knock-in mice. Table 4 shows the approximately 50 protein spots that showed a change in abundance between WT and mutant spots in the 14-day-old lenses. Figure 7 shows 3D plots for some of the protein spots that changed in abundance in the αB-R120G mutant lenses. Heterozygous αB-R120G lenses showed several spots with decreased abundance of phosphoglycerate mutase (spots 5353, 5441, 5456 and 5468). Phosphoglycerate mutase was the only protein in spots 5353 and 5468 but was associated with βB1-crystallin in spots 5441 and 5456. αA- and αB-crystallins decreased in a very basic high molecular weight spot (spot 2982). The abundance of αA-crystallin increased 2.8- to 10-fold in spot 6415, and was slightly degraded and more acidic than normal αA-crystallin. In the same region, spots 6449 and 6848 (αA-crystallin associated with grifin) increased 12-fold and 2.5 fold, respectively. Degraded and more basic forms of αA-crystallin alone (spots 6920 and 7257) or with αB-crystallin and βB3-crystallin (spot 7451) also increased in abundance in heterozygous lenses. A spot containing αA-, γA-, γB-, γC-, and γD-crystallins also decreased 2.7-fold in heterozygous lenses.

Homozygous αB-R120G lenses showed an 8-fold increase in the abundance of a more acidic spot (5961) containing αB- and other crystallins, whereas the more basic spot 5963 decreased 5.6-fold. Spot 6120 containing αA-, αB-, and γB-crystallins also increased in abundance in homozygous lenses. This spot was more acidic than the other αB-crystallin spots and was located near the αA-crystallin position. Spot 5938, which was very close to spot 5963 but slightly more acidic, also decreased in abundance. Spots 7164 increased in abundance by 2.0-fold in αB-R120G homozygous lenses relative to WT. It contained both αA- and αB-crystallins, which were more degraded and basic than the original proteins. Overall, a few unique spots changed in abundance in αB-R120G homozygous lenses than in αB-R120G heterozygous lenses.

To obtain a general perspective of cellular systems affected in the αA-R49C and αB-R120G mutant lenses, we mapped the proteins identified by mass spectrometric analysis to existing networks. These networks represent interactions known to occur among the proteins identified in our analysis. The interactions shown in these networks did not originate from lens tissue in our study. Ingenuity Pathway software analysis generated eight different networks for the proteins identified in the αA-R49C mutant lenses, two of which are shown in Figure 8, with additional

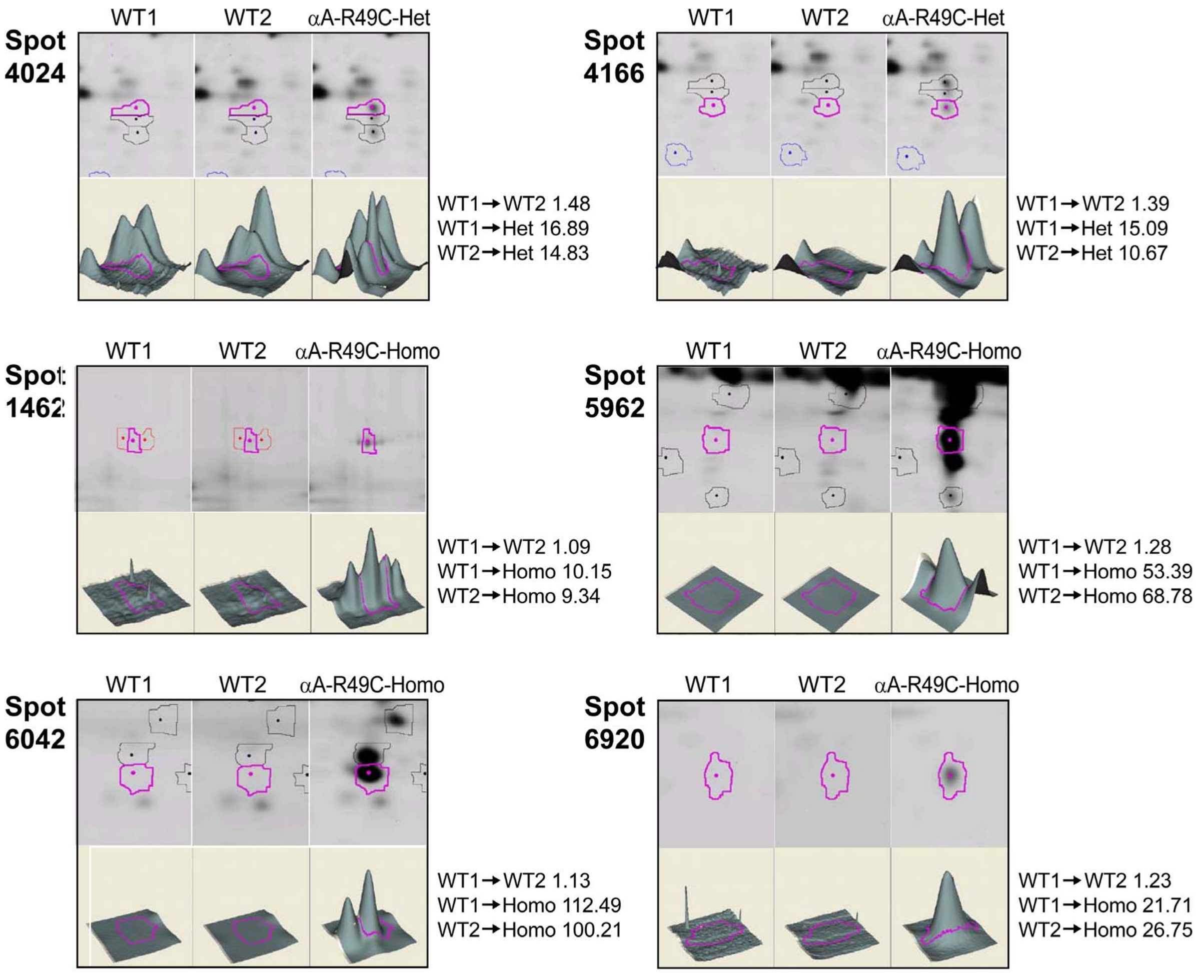

Figure 2. Quantitative analysis of abundance changes in proteins from postnatal 2-day-old WT and αA-R49C knock-in lenses by mass spectrometry. The 3D data sets for representative proteins in two WT (WT1 and WT2) and one αA-R49C heterozygous or αA-R49C homozygous mutant sample are shown. WT1 and WT2 proteins were labeled with Cy2 and Cy3 dyes, respectively and αA-R49C mutant proteins with Cy5. Fold changes between each sample are indicated on the right. See Table 1 for the identity of proteins present in each protein spot.
doi:10.1371/journal.pone.0095507.g002

networks shown in Fig. S3 in File S1. One network generated by this approach included the chaperones HSPA8 and HSPA2 which interact with αB-crystallin. A second network included histone H4 which has been shown to interact with the PI3kinase complex. Four different protein networks were generated by this method in the αB-R120G lenses including one in which the ubiquitin proteasome was at the hub (Figure S4). An interaction between the lens-specific protein grifin and the transcription factor IKZF1 was evident in both αA-R49C and αB-R120G mutant lenses (Figs. S3, S4 in File S1, and Table S2).

Discussion

Several mechanisms can cause hereditary cataracts, including increases in protein mass, aggregation, insolubility, and light scattering. In the present study, we characterized changes in protein abundance at an early postnatal age in mouse lenses with knock-in mutations of αA- or αB-crystallins. We also investigated proteins that showed increased association with αA- or αB-crystallins in mutant lenses, defined by an increase in the level of urea-resistant protein in the same spot.

Several important assumptions of this study require further discussion. The present study identified changes in abundance of many spots in which αA- or αB-crystallin was present together with other proteins. This association indicates similar pI and molecular weights of the ancillary proteins and the α-crystallin in these spots. We cannot speculate on the mechanism by which the proteins are associated with α-crystallins. Our evidence from 2D-gel analysis is suggestive of an association, but is not conclusive. Since this association was observed in multiple gels of wild type and knock-in mutant lenses, the presence of αA- and/or αB-crystallin with specific proteins in the same spots is suggestive of a true association. Previous studies suggest that mutant α-crystallins may exert a gain-of-toxic function on the lens [25]. Thus, it is possible that the differences in protein abundances between normal and knock-in mouse lenses may not be directly due to incompetent chaperones *per se*, although a previous study with the αA and αB-crystallin DKO mouse lenses strengthens the conclusions of the present work [23]. Nevertheless, a toxic gain-of function by the mutant α-crystallins could be a potential factor in the observed results.

There was a significant decrease in the abundance of actin (15.6-fold), filensin (17.5-fold), βA3/A1-crystallin, γD-crystallin (6-fold), and grifin (1.74-fold). We also observed degradation of glutamate dehydrogenase, which was associated with cytochrome

Table 2. Multi-gel analysis of proteins that showed differences in abundance between 2-day-old WT and heterozygous or homozygous αA-R49C lenses.

Spot number	Protein	UNIPROT Accession number	MW (kDa)	Number of assigned spectra	Fold change/*t*-test (*p*-value)		
					WT vs. heterozygous	WT vs. homozygous	Heterozygous/ homozygous
593	Alanyl-tRNA synthetase, cytoplasmic	Q8BGQ7	107	12	2.01/0.035	2.28/0.021	−1.13/0.43
	αA-crystallin	Q569M7	20	2			
	T-complex protein 1 subunit theta	P42932	60	2			
	Glutathione synthetase	P51855	52	2			
884	βA3/A1-crystallin	Q9QXC6	25	1	1.02/0.83	1.65/0.017	−1.62/0.0048
	Putative uncharacterized protein	Q3UAF6	42	1			
928	Heat shock cognate 71 kDa protein	P63017	71	28	1.15/0.11	1.42/0.018	−1.24/0.077
	V-type proton ATPase catalytic subunit	P50516	68	17			
	Stress-70 protein, mitochondrial	P38647	74	5			
	Alpha-fetoprotein	P02772	67	3			
948	Stress-70 protein, mitochondrial	P38647	74	6	1.39/0.064	1.79/0.017	−1.29/0.12
	αA-crystallin	Q569M7	20	4			
	Heat shock cognate 71 kDa protein	P63017	71	3			
	Glutathione synthetase	P51855	52	1			
1081	T-complex protein 1 subunit theta	P42932	60	16	1.93/0.053	2.03/0.044	−1.05/0.43
	αA-crystallin	Q569M7	20	4			
1131	Seryl-tRNA synthetase, cytoplasmic	P26638	58	2	1.39/0.13	1.62/0.051	−1.17/0.04
1146	αA-crystallin	Q569M7	20	9	7.67/0.0037	10.91/0.0018	−1.42/0.075
	T-complex protein 1 subunit theta	P42932	60	3			
1190	βB1-crystallin	Q9WVJ5	28	1	1.1/0.22	1.28/0.013	−1.16/0.029
1351	Filensin	A2AMT1	74	6	1.26/0.006	1.22/0.014	1.03/0.68
	Eukaryotic translation initiation factor	P60229	52	3			
1477	αA-crystallin	Q569M7	20	5	−3.53/0.063	−4.25/0.043	1.2/0.47
	βA3/A1-crystallin	Q9QXC6	25	2			
1519	Creatine kinase B- type	Q04447	43	11	15.85/0.00026	22.33/0.0001	−1.41/0.14
	αA-crystallin	Q569M7	20	9			
	Putative uncharacterized protein	Q3UAF6	42	2			
1540	αA-crystallin	Q569M7	20	11	53.41/6.9e-005	79.7/5.10E-05	−1.49/0.077
1582	αA-crystallin	Q569M7	20	13	10.11/0.0025	14.62/0.0014	−1.45/0.098
1612	αA-crystallin	Q569M7	20	9	−2.77/0.12	−4.82/0.041	1.74/0.19
1625	αA-crystallin	Q569M7	20	1	2.75/0.031	−1.42/0.21	3.9/0.01
	Fructose-biphosphate aldolase A	P05064	36	1			
1659	αA-crystallin	Q569M7	20	12	1.44/0.0027	2.24/0.0069	−1.56/0.12
	Eukaryotic translation initiation factor	Q9QZD9	36	2			

Table 2. Cont.

Spot number	Protein	UNIPROT Accession number	MW (kDa)	Number of assigned spectra	Fold change/*t*-test (*p*-value)		
					WT vs. heterozygous	WT vs. homozygous	Heterozygous/ homozygous
	Putative uncharacterized protein	Q3UAF6	42	1			
1767	Glyceraldehyde-3-phosphate dehydrogenase	P16858	36	2	−2.11/0.085	−1.78/0.069	−1.19/0.12
	Heterogeneous nuclear ribonucleoproteins A2/B1	O88569	37	2			
1786	Heterogeneous ribonucleoprotein A1	P49312	34	5	−1.93/0.053	−1.16/0.42	−1.67/0.088
1856	βB1-crystallin	Q9WVJ5	28	8	1.08/0.54	−1.65/0.089	1.77/0.0045
1865	βB1-crystallin	Q9WVJ5	28	13	−2.14/0.0017	−1.42/0.075	−1.51/0.057
1868	βB1-crystallin	Q9WVJ5	28	8	−1.04/0.47	−1.48/0.049	1.42/0.12
	Eukaryotic translation initiation factor 4H	Q9WUK2	27	2			
1902	βB1-crystallin	Q9WVJ5	28	12	1.06/0.50	−2/0.0075	2.13/0.0022
2640	αA-crystallin	Q569M7	20	9	−2.15/0.00096	−2.66/0.00022	1.24/0.036
	Histone H4	P62806	11	2			
	Implantin (fragment)	P83891	2	1			
2661	Regulating synaptic membrane exocytosis protein 2	Q9EQZ7	173	1	−2.38/0.039	1.00/0.95	−2.39/0.021
2684	Myotrophin	P62774	13	2	−1.32/0.11	−1.57/0.033	1.19/0.16
2772	αA-crystallin	Q569M7	20	8	−3.65/0.074	−7.3/0.03	2/0.18
	βA4-crystallin	Q9JJV0	22	3			
	βA3/A1-crystallin	Q9QXC6	25	2			
1923	βB1-crystallin	Q9WVJ5	28	2	1.43/0.053	−1.54/0.019	2.2/0.011
2018	αA-crystallin	Q569M7	20	8	−1.4/0.021	−1.13/0.35	−1.24/0.059
	Implantin (fragment)	P83891	2	2			
	Coactosin-like protein	Q9CQI6	16	2			
2109	βA3/A1-crystallin	Q9QXC6	25	12	−1.16/0.18	−1.49/0.034	1.28/0.15
	αA-crystallin	Q569M7	20	6			
	βA4-crystallin	Q9JJV0	22	5			
	βA2-crystallin	Q9JJV1	22	2			
2115	βA3/A1-crystallin	Q9QXC6	25	9	−3.14/0.059	−3.92/0.034	1.25/0.48
	αB-crystallin	P23296	20	9			
	βB2-crystallin	P62696	23	9			
	βB3-crystallin	Q9JJU9	24	8			
	βA2-crystallin	Q9JJV1	22	8			
	αA-crystallin	Q569M7	20	6			

Table 2. Cont.

Spot number	Protein	UNIPROT Accession number	MW (kDa)	Number of assigned spectra	Fold change/*t*-test (*p*-value)		
					WT vs. heterozygous	WT vs. homozygous	Heterozygous/ homozygous
2123	αA-crystallin	Q569M7	20	17	−4.44/0.029	−8.57/0.016	1.93/0.17
	βA3/A1-crystallin	Q9QXC6	25	8			
	βA2-crystallin	Q9JJV1	22	8			
	βB2-crystallin	P62696	23	6			
	αB-crystallin	P23296	20	3			
	βB3-crystallin	Q9JJU9	24	2			
2154	αA-crystallin	Q569M7	20	12	−1.44/0.0024	−1.26/0.0094	−1.14/0.13
2233	αB-crystallin	P23296	20	19	−1.59/0.084	−1.8/0.053	1.13/0.35
	γD-crystallin	Q6PGI0	20	16			
	γA-crystallin	P04345	21	7			
	γF-crystallin	Q9CXV3	21	4			
	γB-crystallin	Q6PHP7	21	3			
	βA3/A1-crystallin	Q9QXC6	25	2			
	Plectin	Q9QXS1	534	2			
2261	γA-crystallin	P04345	21	2	1.65/0.074	−1.53/0.11	2.52/0.022
	αB-crystallin	P23296	20	2			
2294	αA-crystallin	Q569M7	20	16	4.16/0.020	5.64/0.012	−1.36/0.054
2317	αA-crystallin	Q569M7	20	14	−2.83/0.095	−5.09/0.032	1.8/0.17
2320	αA-crystallin	Q569M7	20	21	−4.85/0.11	−9.28/0.035	1.91/0.14
2337	βA3/A1-crystallin	Q9QXC6	25	8	−2.28/0.024	−3.49/0.06	1.53/0.23
	αA-crystallin	Q569M7	20	8			
	βA2-crystallin	Q9JJV1	22	2			
2351	αA-crystallin	Q569M7	20	12	4.61/0.0057	10.45/0.0049	−2.27/0.14
2388	αA-crystallin	Q569M7	20	10	1.48/0.27	5.71/0.038	−3.85/0.074
2413	γD-crystallin	Q6PGI0	20	10	−1.14/0.86	−3.8/0.046	3.33/0.007
	Peptidyl-prolyl cis-trans isomerase	P17742	18	6			
	γA-crystallin	P04345	21	6			
	γB-crystallin	Q6PHP7	21	4			
	γC-crystallin	Q61597	21	4			
2417	αA-crystallin	Q569M7	20	15	7.51/0.0023	10.53/0.0015	−1.4/0.15
2454	Nucleoside diphosphate kinase	E9PZF0	30	6	−1.25/0.016	−1.47/0.0067	1.18/0.11
	Peptidyl-prolyl cis-trans isomerase	P17742	18	4			
	γD-crystallin	Q6PGI0	20	3			
	αA-crystallin	Q569M7	20	3			

Table 2. Cont.

Spot number	Protein	UNIPROT Accession number	MW (kDa)	Number of assigned spectra	Fold change/*t*-test (*p*-value)		
					WT vs. heterozygous	WT vs. homozygous	Heterozygous/ homozygous
	γB-crystallin	Q6PHP7	21	2			
2462	αA-crystallin	Q569M7	20	3	−1.48/0.12	−2.08/0.017	1.41/0.021
	γN-crystallin	Q8VHL5	21	2			
2469	Nucleoside diphosphate kinase	E9PZF0	30	6	−1.37/0.052	−1.56/0.023	1.14/0.17
	αA-crystallin	Q569M7	20	3			
2501	γC-crystallin	Q61597	21	2	−1.55	−2.11/0.012	1.36
2533	αA-crystallin	Q569M7	20	3	−1.82/0.10	−2.44/0.027	1.34/0.26
2538	αA-crystallin	Q569M7	20	3	−2.41/0.021	−2.32/0.067	−1.04/0.88
	40S ribosomal protein S12	P63323	15	2			
2553	Fatty acid-binding protein epidermal	Q05816	15	12	−1.96/0.67	−3.48/0.2	1.77/0.19
	αA-crystallin	Q569M7	20	7			
2631	αA-crystallin	Q569M7	20	2	−2.58/0.13	−2.92/0.038	1.13/0.079

WT, wild-type.

doi:10.1371/journal.pone.0095507.t002

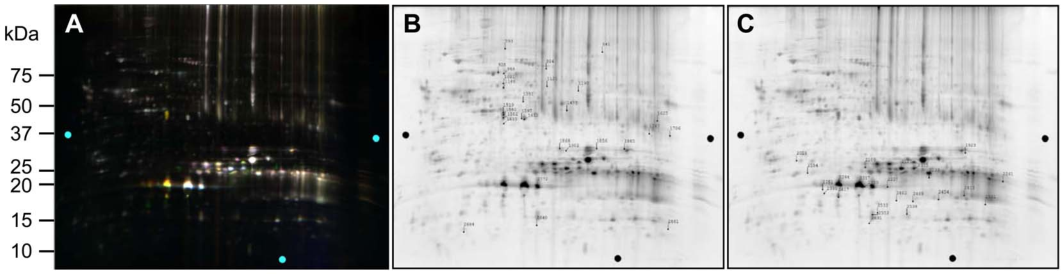

Figure 3. 2D-DIGE analysis of proteomic changes in whole lenses of WT, αA-R49C heterozygous, and αA-R49C homozygous mutant lenses using a pool-based analysis. (A) WT samples were labeled with Cy2, a pool of all samples (containing WT, αA-R49C heterozygous and homozygous proteins) was labeled with Cy3, and the αA-R49C heterozygous mutant sample was labeled with Cy5. The pool sample was a common comparator for each sample. (B, C) Spots that were selected based on analysis of the gels are shown. Quantitative image analysis by biological variation analysis was performed across several samples, and mass spectrometry data for the identified proteins from these gels are listed in Table 3.
doi:10.1371/journal.pone.0095507.g003

c in some spots. Because the abundance of these proteins changed at a young age, even in the heterozygous mutant αA-R49C lens, with no apparent change in lens morphology, it is very likely that they are *in vivo* substrates of α-crystallin. Our analysis also suggests that enzymes involved in lens metabolism, such as creatine kinase B and phosphoglycerate mutase, and the detoxification enzyme GST-μ are *in vivo* substrates of αA- and αB-crystallins. These proteins may be structurally labile and might interact with αA- and αB-crystallins for conformational maintenance during the early stages of lens growth but become more stably associated with the protein when the chaperone is mutated. Structural analysis of these enzymes is necessary to reveal any common structural domains. These findings suggest that key metabolic pathways are involved in the mechanism of cataract formation by the αA-R49C or αB-R120G mutations. The decrease in phosphoglycerate mutase levels in the postnatal αB-R120G knock-in mouse lens suggests that mutation of the chaperone protein in the lens affects lens metabolism even before the opacification process becomes evident.

The association of histones with αA-crystallin increased in the mutant lenses. The possibility that histones are protected by α-crystallins is particularly important because histones are critical and long-lived proteins [26]. The R49C mutant of αA-crystallin exhibits increased apoptosis and aberrant accumulation of nuclei in the lens, suggesting a possible explanation for the increased abundance of histones [15,27]. We previously reported an increased abundance of histones in αA/αB double knock-out (DKO) lenses [23], and in lens cells expressing another human cataract-related mutant of αA-crystallin in which the arginine 116 residue is replaced by cysteine [28]. Therefore it seems likely that histones may be protected by αA- and αB-crystallins in the lens. In 2-week-old αA-R49C mutant lenses there was an increase in αA-crystallin associated with annexins. These proteins are associated with apoptosis, which has been observed in the αA-R49C mouse. Interestingly, phosphoglycerate mutase, α-enolase, and peptidyl-prolyl cis-trans isomerase are oxidized and have reduced enzyme activities in Alzheimer's disease, another disease associated with protein aggregation [29].

An intriguing observation of the present study was the presence of albumin in the 2-day-old lens (Table 1). Extracellular albumin, an abundant protein in the aqueous humor, becomes internalized in the lens in vivo [30]. It has been suggested that albumin is a carrier for lipids and other metabolites, and could be essential for normal lens physiology [31,32]. A decrease in plasma albumin has been linked with an increased risk of human cataract [33]. The abundance of the spot containing albumin, αA-crystallin and filensin showed a 3.6-fold variation between the two biological replicates of the WT mouse lens, and increased 16- to 17-fold in the αA-R49C heterozygous lenses. Further studies will be necessary to understand the significance of these observations. We detected increased αA-crystallin in protein spots containing cytoskeletal proteins, and increased abundance of degraded and more acidic cytoskeletal proteins including spectrin-α, filensin, phakinin, tubulin, vimentin, and microtubule-associated protein RP/EB in the αA-R49C mutant knock-in lenses. The abundance of filensin and phakinin decreased in αA-R49C mutant lenses, suggesting that these proteins are *in vivo* substrates for αA-crystallin. The spectrin-actin membrane skeleton contributes significantly to lens fiber cell organization and is functionally linked to the phakinin-filensin network [34]. Disruption of the spectrin-actin membrane cytoskeletal complexes may therefore be responsible for the morphological changes observed in αA-R49C homozygous mutant lenses at an early age [27,35]. There was also an increase in the amount of degraded and more acidic grifin, a protein whose interaction with αA-crystallin has been demonstrated previously [36], and the abundance of αA-R49C associated with grifin increased 16-fold in homozygous mutant lenses. The amount of hemoglobin subunit α decreased in αA-R49C homozygous mutant lenses indicating that it is a likely substrate for αA-crystallin. Previous studies support the possibility that destabilized forms of hemoglobin show increased binding to αB-crystallin *in vitro* [37].

We found an increase in β-crystallin isoforms with more acidic pI in the mutant lenses. Decreases in more basic forms of βB1- and βB3-crystallins and increases in more acidic forms indicate that αA-crystallin is a chaperone for these two crystallins. Furthermore, αA- and αB-crystallins were increasingly associated with β-crystallins in the mutant lenses, suggesting that they may have formed heteromeric complexes. Previous studies have identified covalent multimers of crystallins in aging human lenses [38]. Recently, the crosslinks between β-crystallin isoforms have been identified by mass spectrometry [39]. Deamidation of βB2-crystallin has been proposed to disrupt normal crystallin structure and short-range order necessary for lens transparency [40]. Deamidation has been shown to lower the temperature necessary for βB2-crystallin unfolding and aggregation, suggesting decreased βB2-crystallin stability, although its 3D dimeric structure was not significantly altered [41]. Interestingly, the nature and amount of the destabilized β-crystallin intermediate is important for recognition by the chaperone [42]. Decreased amounts of βB1-crystallin were detected in five spots and in an additional four spots containing other β-crystallin polypeptides. αA-crystallin was associated with β-crystallins in these spots. The decrease in βB1-crystallin was noteworthy because βB1-crystallin has a unique role

Table 3. Protein spots that showed a change in abundance between 2-week-old WT and αA-R49C homozygous mutant lenses.

Spot number	Protein	Uniprot accession number	MW (kDa)	Number of assigned spectra	Fold change		
					WT1 vs. WT2	WT1 vs. homozygous	WT2 vs. homozygous
700	Spectrin α-2	A3KGU5	283	47	−1.6	−13.77	−8.51
769	Spectrin α-2	A3KGU5	283	20	1.77	−8.19	−14.35
	γA-crystallin		21	2			
1164	Adult male sm. intestine cDNA	Q9CPN9	26	1	−2.56	−7.87	−3.05
2448	Filensin	A2AMT1	74	39	−2.05	−15.14	−7.32
2675	Filensin	A2AMT1	74	8	−1.89	3.81	7.27
	78 kDa glucose-regulated protein precursor	P20029	72	4			
	GTP binding protein Di- Ras 1	Q91Z61	22	1			
	Collagen α2v chain precursor	Q3U962	145	1			
2676	78 kDa glucose-regulated protein precursor	P20029	72	12	−1.87	3.4	6.42
3641	Phakinin	Q6NVD9	46	24	−1.59	−10.22	−6.38
3880	Phakinin	Q6NVD9	46	15	1.23	8.33	6.82
	16 days embryo kidney cDNA	Q3TGJ9	81	7			
4035	αA-crystallin	Q569M7	20	8	1.00	7.71	7.76
	βA3/A1-crystallin	Q9QXC6	25	4			
	βB1-crystallin	Q9WVJ5	28	2			
	βB3-crystallin	Q9JJU9	24	2			
4058	βA3/A1-crystallin	Q9QXC6	25	7	1.77	3.79	2.16
	αA-crystallin	Q569M7	20	4			
	βB1-crystallin	Q9WVJ5	28	1			
	Fructose-bisphosphate aldolase	A6ZI44	45	1			
4101	αA-crystallin	Q569M7	20	8	1.2	3.41	2.86
	βA3/A1-crystallin	Q9QXC6	25	6			
	βB3-crystallin	Q9JJU9	24	2			
	βB1-crystallin	Q9WVJ5	28	1			
4111	αA-crystallin	Q569M7	20	3	−1.49	−10.27	−6.85
	βA3/A1-crystallin	Q9QXC6	25	3			
	βB3-crystallin	Q9JJU9	24	2			
	40S ribosomal protein SA(Laminin receptor 1)	P14206	33	1			
4191	αA-crystallin	Q569M7	20	7	−1.07	26.32	28.36
	βB1-crystallin	Q9WVJ5	28	2			
4207	Phakinin	Q6NVD9	46	8	2.02	−9.58	−19.19
	αA-crystallin	Q569M7	20	1			

Table 3. Cont.

Spot number	Protein	Uniprot accession number	MW (kDa)	Number of assigned spectra	Fold change		
					WT1 vs. WT2	WT1 vs. homozygous	WT2 vs. homozygous
4217	αA-crystallin	Q569M7	20	10	1.36	5.67	4.21
	βA3/A1-crystallin	Q9QXC6	25	7			
	βB1-crystallin	Q9WVJ5	28	2			
	Phakinin	Q6NVD9	46	2			
4627	αA-crystallin	Q569M7	20	8	1.21	22.44	18.67
	βA3/A1-crystallin	Q9QXC6	25	2			
	Adult male stomach cDNA	Q3Y2E0	27	2			
	Adult male sm. intestine cDNA	Q9CPN9	26	1			
	WD repeat-containing protein 81	Q5ND34	96	1			
	NOD-derived CD11c positive dendritic cells cDNA	Q5ND34	17	1			
4872	αA-crystallin	Q569M7	20	5	−1.31	3.99	5.26
	Annexin A5	ANXA5	36	5			
	Adult male stomach cDNA	Q3Y2E0	27	2			
	Adult male small intestine cDNA	Q9CPN9	26	1			
	SRC kinase signaling inhibitor 1	Q9QWI6	135	1			
	Filamin-B	Q80X90	278	1			
	Probable E3 ubiquitin-protein ligase HERC2	Q4U2R1	528	1			
5218	βB1-crystallin	Q9WVJ5	28	13	1.76	−24.55	−42.86
5225	βB1-crystallin	Q9WVJ5	28	20	−1.05	−6.09	−5.72
5236	βB1-crystallin	Q9WVJ5	28	20	−1.27	−8.76	−6.82
	Complement factor C2	Q792Q3	53	1			
5608	βB1-crystallin	Q9WVJ5	28	15	−1.23	−8.75	−7.05
	αA-crystallin	Q569M7	20	1			
5609	βB3-crystallin	Q9JJU9	24	19	1.43	21.18	14.97
	Glutathione S-transferase μ	A2AE89	24	5			
	βB2-crystallin	P62696	23	3			
	βB1-crystallin	Q9WVJ5	28	1			
	Complement factor C2	Q792Q3	53	1			
	Adult male sm. intestine cDNA	Q9CPN9	26	1			
5617	βB2-crystallin	P62696	23	34	−1.55	−8.44	−5.38
	βB3-crystallin	Q9JJU9	24	12			
	Glutathione S-transferase μ	A2AE89	24	5			

Table 3. Cont.

Spot number	Protein	Uniprot accession number	MW (kDa)	Number of assigned spectra	Fold change		
					WT1 vs. WT2	WT1 vs. homozygous	WT2 vs. homozygous
	βA3/A1-crystallin	Q9QXC6	25	2			
	βB1-crystallin	Q9WVJ5	28	1			
5625	βB2-crystallin	P62696	23	17	−1.38	−18.58	−13.36
	βB3-crystallin	Q9JJU9	24	6			
	Heat shock protein B1 (HspB1)	P14602	23	2			
	Glutathione S-transferase μ	A2AE89	24	2			
	NOD-derived Cd11c-positive dendritic cells cDNA	Q3TCH2	25	2			
5695	βA3/A1-crystallin	Q9QXC6	25	66	−1.53	−20.2	−13.04
	βB2-crystallin	P62696	23	56			
	βB3-crystallin	CRBB3	24	13			
	START domain containing 9	AZAKH9	408	1			
5719	βB2-crystallin	P62696	23	46	1.23	8.38	6.87
	βA3/A1-crystallin	Q9QXC6	25	36			
	βB3-crystallin	CRBB3	24	32			
	βB1-crystallin	CRBB1	28	10			
	αA-crystallin	Q569M7	20	4			
5753	βB3-crystallin	CRBB3	24	67	1.49	9.82	6.67
	βB2-crystallin	P62696	23	29			
	βA3/A1-crystallin	Q9QXC6	25	9			
5789	βB3-crystallin	CRBB3	24	40	1.02	17.66	17.52
	βB1-crystallin	CRBB1	28	21			
	βB2-crystallin	P62696	23	6			
	αA-crystallin	Q569M7	20	4			
	Development and differentiation enhancing factor 2	Q7SIG6	107	3			
	βA3/A1-crystallin	Q9QXC6	25	2			
5799	βB1-crystallin	CRBB1	28	38	−1.1	13.89	15.37
	βA3/A1-crystallin	Q9QXC6	25	11			
	Proteasome subunit beta type 4	P99026	29	6			
	αA-crystallin	Q569M7	20	3			
	βA4-crystallin	Q9JJV0	22	3			
	Development and differentiation enhancing factor 2	Q7SIG6	107	2			

Table 3. Cont.

Spot number	Protein	Uniprot accession number	MW (kDa)	Number of assigned spectra	Fold change		
					WT1 vs. WT2	WT1 vs. homozygous	WT2 vs. homozygous
	Adult male small intestine cDNA	Q9CPN9	26	2			
	Prickle3 protein	Q8CIL5	72	1			
	βB3-crystallin	CRBB3	24	1			
	Cardiotrophin-like cytokine factor 1 precursor	Q9QZM3	25	1			
	βA2-crystallin	CRBA2	22	1			
	UTP14, U3 small nucleolar ribonucleoprotein homolog A yeast	Q4QY64	87	1			
	Leucine-rich repeat-containing protein 33 precursor	Q8BMT4	77	1			
	Adult male testis cDNA RIKEN	Q9D9G7	28	1			
	GTP-binding protein Di-Ras1	Q91Z61	22	1			
	NOD-derived CD11c positive dendritic cells cDNA	Q8VDD8	55	1			
	16 days neonate thymus cDNA	Q3TRH2	48	1			
	Bone marrow macrophage cDNA	Q3UAB1_MOUSE	50	1			
	DNA polymerase epsilon subunit 2	DPOE2_MOUSE	59	1			
5830	βB1-crystallin	CRBB1	28	29	1.19	−8.59	−10.09
	βS (γS-crystallin)	O35486	21	15			
	γC-crystallin	Q61597	21	14			
	γB-crystallin	Q6PHP7	21	10			
	βA3/A1-crystallin	Q9QXC6	25	4			
	γD-crystallin	Q6PGI0	21	4			
	Glutathione S-transferase P1	P19157	24	3			
	αA-crystallin	Q569M7	20	2			
6012	αB-crystallin	P23927	20	42	1.07	14.08	13.3
	γB-crystallin	Q6PHP7	21	29			
	βA2-crystallin	CRBA2	22	25			
	βA3/A1-crystallin	Q9QXC6	25	23			
	βS (γS-crystallin)	O35486	21	20			
	γN-crystallin	Q8VHL5	21	18			
	αA-crystallin	Q569M7	20	15			
	γA-crystallin	P04345	21	8			
	γD-crystallin	Q6PGI0	21	7			
	γC-crystallin	Q61597	21	3			

Table 3. Cont.

Spot number	Protein	Uniprot accession number	MW (kDa)	Number of assigned spectra	Fold change		
					WT1 vs. WT2	WT1 vs. homozygous	WT2 vs. homozygous
6117	αB-crystallin	P23927	20	43	−1.14	4.51	5.17
	γB-crystallin	Q6PHP7	21	24			
	βA3/A1-crystallin	Q9QXC6	25	17			
	βA2-crystallin	CRBA2	22	14			
	γA-crystallin	P04345	21	11			
	γD-crystallin	Q6PGI0	21	10			
	γS-crystallin	O35486	21	7			
	αA-crystallin	Q569M7	20	4			
	γN-crystallin	Q8VHL5	21	4			
	γC-crystallin	Q61597	21	3			
	γE-crystallin	A2RTH4	21	3			
	Sodium channel voltage-gated Type III alpha	A2ASI5	221	1			
6161	αB-crystallin	P23927	20	30	1.61	10.3	6.45
	αA-crystallin	Q569M7	20	19			
	γB-crystallin	Q6PHP7	21	6			
	βA3/A1-crystallin	Q9QXC6	25	6			
	γD-crystallin	Q6PGI0	21	5			
	βA2-crystallin	CRBA2	22	4			
	γA-crystallin	P04345	21	3			
	Tenascin precursor	Q80YX1	232	2			
	Mark 1 protein	Q14DQ3	88	1			
	Tenascin precursor	Q80YX1	232	1			
6341	αA-crystallin	Q569M7	20	31	1.2	−60.51	−71.83
	αB-crystallin	P23927	20	9			
	βA3/A1-crystallin	Q9QXC6	25	6			
	βA2-crystallin	CRBA2	22	5			
	γN-crystallin	Q8VHL5	21	5			
	βB1-crystallin	CRBB1	28	4			
	βA4-crystallin	Q9JJV0	22	3			
	γA-crystallin	P04345	21	2			
6352	αA-crystallin	Q569M7	20	25	1.2	−13.31	−15.81
	αB-crystallin	P23927	20	14			
	βA3/A1-crystallin	Q9QXC6	25	7			

Table 3. Cont.

Spot number	Protein	Uniprot accession number	MW (kDa)	Number of assigned spectra	Fold change		
					WT1 vs. WT2	WT1 vs. homozygous	WT2 vs. homozygous
	Adult male small intestine cDNA	Q9CPN9	26	3			
	Cardiotrophin-like cytokine factor 1 precursor	Q9QZM3	25	3			
	Leucine-rich repeat-containing protein 33 precursor	Q8BMT4	77	2			
	ELMO domain-containing protein 1	Q3V1U8	38	2			
	γA-crystallin	P04345	21	1			
	UTP14, U3 small nucleolar ribonucleoprotein homolog A yeast	Q4QY64	87	1			
	βA2-crystallin	CRBA2	22	1			
	Adult male testis cDNA RIKEN	Q9D9G7	28	1			
	GTP-binding protein Di-Ras 1	Q91Z61	22	1			
	Regulator of nonsense transcripts 1	Q9EPU0	124	1			
6485	αA-crystallin	Q569M7	20	26	1.35	34.08	25.47
	Adult male small intestine cDNA	Q9CPN9	26	2			
	αB-crystallin	P23927	20	2			
	Adult male testis cDNA RIKEN	Q9D9G7	28	2			
	γA-crystallin	P04345	21	1			
	Pyruvate carboxylase, mitochondrial	Q05920	130	1			
	GTP-binding protein Di-Ras 1	Q91Z61	22	1			
	ATPase family AAA domain-containing protein 5 (chromosome fragility-associated gene 1 protein	Q4QY64	204	1			
	MKIAA0327 splice variant	Q8CHE5	102	1			
6520	γA-crystallin	P04345	21	32	1.22	−5.96	−7.23
	γB-crystallin	Q6PHP7	21	22			
	γC-crystallin	A3RLD4	21	19			
	γD-crystallin	Q6PGI0	21	8			
	Adult male small intestine cDNA	Q9CPN9	26	1			
6663	αA-crystallin	Q569M7	20	35	1.75	15.24	8.8
	γC-crystallin	A3RLD4	21	11			
	γA-crystallin	P04345	21	8			
	γB-crystallin	Q6PHP7	21	7			
	2 days neonate thymus thymic cells cDNA	Q7TNP7	69	3			
	Adult male small intestine cDNA	Q9CPN9	26	2			

Table 3. Cont.

Spot number	Protein	Uniprot accession number	MW (kDa)	Number of assigned spectra	Fold change		
					WT1 vs. WT2	WT1 vs. homozygous	WT2 vs. homozygous
	γD-crystallin	Q6PGI0	21	2			
	Nuclear protein localization protein 4 homolog	P60670	68	2			
6668	αA-crystallin	Q569M7	20	28	1.72	73.82	43.31
	Adult male small intestine cDNA	Q9CPN9	26	2			
	2 days neonate thymus thymic cells cDNA	Q7TNP7	69	2			
	Nuclear protein localization protein 4 homolog	P60670	68	2			
	Adult male testis cDNA RIKEN	Q9D9G7	28	2			
	ELMO domain-containing protein 1	Q3V1U8	38	2			
	Prickle3 protein	Q8CIL5	72	2			
6788	αA-crystallin and many others	Q569M7	20	30	2.57	498.57	195.82
	Nuclear protein localization protein 4 homolog	P60670	68	3			
	2 days neonate thymus thymic cells cDNA	Q7TNP7	69	2			
7068	αA-crystallin	Q569M7	20	27	−1.05	−21.14	−20.03
	2 days neonate thymus thymic cells cDNA	Q7TNP7	69	2			
	Prickle3 protein	Q8CIL5	72	2			
	γA-crystallin	P04345	21	2			
7089	γS-crystallin	O35486	21	14	1.76	−8.63	−15.01
	γC-crystallin	A3RLD4	21	8			
	γB-crystallin	Q6PHP7	21	3			
	αA-crystallin	Q569M7	20	2			
	γD-crystallin	Q6PGI0	21	1			
7269	αA-crystallin	Q569M7	20	22	1.1	55.72	51.25
	UTP14, U3 small nucleolar ribonucleoprotein homolog A yeast	Q4QY64	87	2			
	Palmitoyl protein thioesterase-like protein	Q8R2F8	16	1			
7419	αA-crystallin	Q569M7	20	18	−2.33	14.04	32.99
	UTP14, U3 small nucleolar ribonucleoprotein homolog A yeast	Q4QY64	87	2			
	2 days neonate thymus thymic cells cDNA	Q7TNP7	69	2			
	Adult male small intestine cDNA	Q9CPN9	26	2			
7540	αA-crystallin	Q569M7	20	20	1.02	91.8	91.26

Table 3. Cont.

Spot number	Protein	Uniprot accession number	MW (kDa)	Number of assigned spectra	Fold change		
					WT1 vs. WT2	WT1 vs. homozygous	WT2 vs. homozygous
	Ceruloplasmin precursor	Q61147	121	8			
	Heparin cofactor 2 precursor	HEP2_mouse	54	4			
	Plexin-A4 precursor	Q80UG2	213	3			
	Attractin	Q9WU60	158	3			
	Serum Amyloid-P component	Q4JFI8	26	3			
	Lumican precursor	P51885	38	2			
	Gelsolin	P13020	86	2			
	UTP14, U3 small nucleolar ribonucleoprotein homolog A yeast	Q4QY64	87	2			
	ELMO domain-containing protein 1	Q3V1U8	38	2			
	Complement factor I precursor	Q61129	67	2			
7568	αA-crystallin	Q569M7	20	17	−1.45	134.39	197.02
	Ceruloplasmin precursor	Q61147	121	9			
	Gelsolin	P13020	86	4			
	Lumican precursor	P51885	38	4			
	Pyruvate carboxylase, mitochondrial	Q05920	130	3			
	Plexin-A4 precursor	Q80UG2	213	3			
	ELMO domain-containing protein 1	Q3V1U8	38	3			
	Complement factor I precursor	Q61129	67	3			
	Cardiotrophin-like cytokine factor 1 precursor	Q9QZM3	25	2			
	GTP-binding protein Di-Ras 1	Q91Z61	22	2			
	Myoferlin	Q69ZN7	233	2			
7751	αA-crystallin	Q569M7	20	9	1.15	160.65	141.07
	Ceruloplasmin precursor	Q61147	121	8			
	Lumican precursor	P51885	38	4			
	ELMO domain-containing protein 1	Q3V1U8	38	2			
	Cardiotrophin-like cytokine factor 1 precursor	Q9QZM3	25	2			
8192	αA-crystallin	Q569M7	20	7	1.24	75.08	61.08
	Ceruloplasmin precursor	Q61147	121	7			
	Lumican precursor	P51885	38	3			
	ELMO domain-containing protein 1	Q3V1U8	38	3			
	Adult male testis cDNA RIKEN	Q9D9G7	28	3			
	Adult male small intestine cDNA	Q9CPN9	26	2			

Table 3. Cont.

Spot number	Protein	Uniprot accession number	MW (kDa)	Number of assigned spectra	Fold change		
					WT1 vs. WT2	WT1 vs. homozygous	WT2 vs. homozygous
	UTP14, U3 small nucleolar ribonucleoprotein homolog A yeast	Q4QY64	87	2			

WT, wild-type.
doi:10.1371/journal.pone.0095507.t003

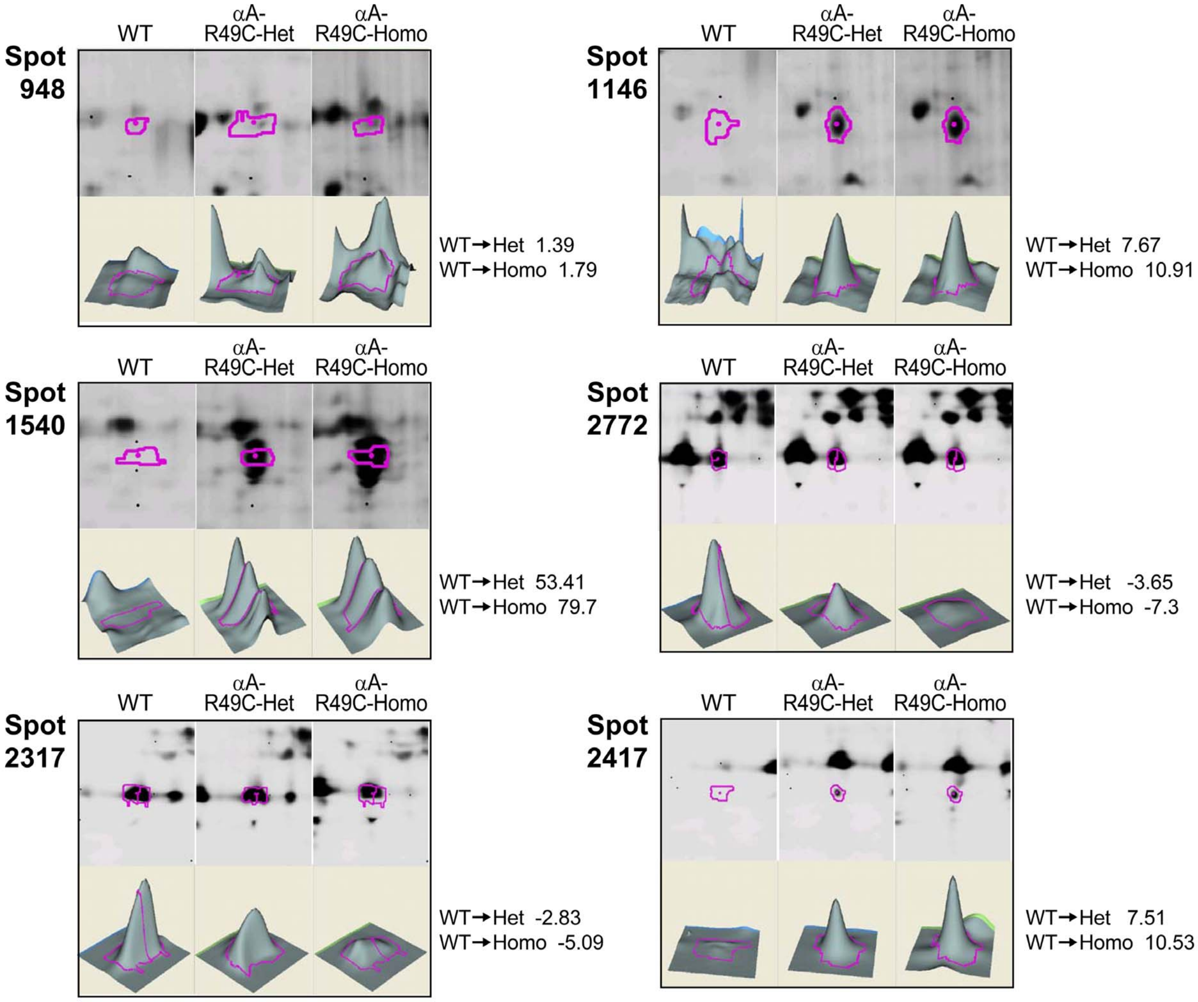

Figure 4. Pool-based quantitative analysis of changes in abundance of postnatal 2-day-old lens proteins from WT and αA-R49C knock-in lenses by mass spectrometry. The 3D data sets for representative proteins in one WT, one pool, and one αA-R49C heterozygous or αA-R49C homozygous mutant sample are shown. WT proteins were labeled with Cy2, pool proteins with Cy3, and αA-R49C heterozygous mutant proteins with Cy5. Fold changes between each sample are indicated on the right. See Table 2 for the identity of proteins present in each protein spot.
doi:10.1371/journal.pone.0095507.g004

in promoting higher order crystallin association in the lens, and any change in this order could result in increased light scattering and loss of transparency [43–45]. The amount of αA and αB-crystallins associating with βA3/A1-, βA2-, and βA4-crystallins increased significantly in homozygous 2-day-old lenses. Our studies also demonstrated a decrease in γ-crystallins in homozygous lenses at a young age. Many of these changes occurred in a mutation- and dose-dependent manner; i.e., changes in the

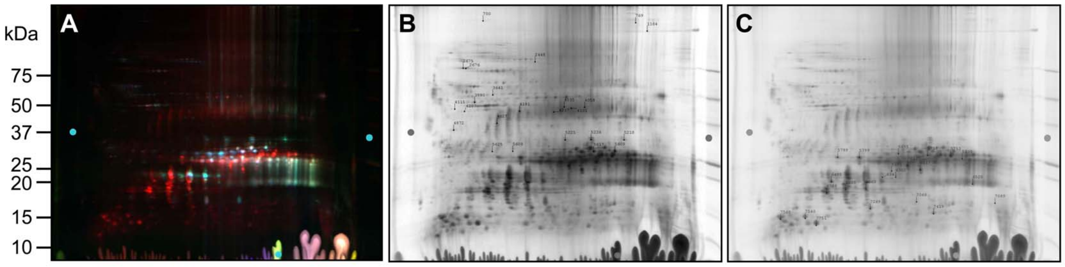

Figure 5. 2D-DIGE analysis of proteomic changes in whole lenses of 14-day-old mice induced by knock-in of the αA-R49C mutation. (A) A 2D gel of lens proteins labeled with cyanine dyes derived from WT1 proteins labeled with Cy2, WT2 proteins labeled with Cy3, and αA-R49C homozygous lens proteins labeled with Cy5. (B, C) Protein spots that were selected for analysis from the gel in (A) are shown. Proteins were identified by tandem mass spectrometry and Mascot searches of spots that were selected from the gels. Quantitative image analysis and mass spectrometry data for the identified proteins from these gels are listed in Table 3.
doi:10.1371/journal.pone.0095507.g005

Table 4. Quantitative analysis of protein abundance in 14-day-old WT, αB-R120G lenses heterozygous and homozygous.

Spot number	Protein	UNIPROT accession number	MW (kDa)	Number of assigned spectra	WT1 vs. WT2	WT1 vs. heterozygous	WT2 vs. heterozygous
2982	αB-crystallin	P23927	20	2	1.19	−1.82	−2.34
	αA-crystallin	Q569M7	20	1			
	RIKEN cDNA 2210010C04, isoform CRA_b	Q9CPN9	26	1			
	Probable peptide chain release factor C12orf65 homolog, mitochondrial	Q80VP5	21	1			
4441	αA-crystallin	Q569M7	20	1	1.13	−2.03	−2.48
5353	Phosphoglycerate mutase	O70250	29	2	1.15	−2.06	−2.55
5432	βB1-crystallin	Q9WVJ5	28	17	1.06	−1.81	−2.06
	βB3-crystallin	Q9JJU9	24	5			
	αA-crystallin	Q569M7	20	1			
	αB-crystallin	P23927	20	1			
5441	βB1-crystallin	Q9WVJ5	28	2	1.06	−1.93	−2.2
	Phosphoglycerate mutase	O70250	29	2			
5456	βB1-crystallin	Q9WVJ5	28	5	1.22	−1.94	−2.56
	Phosphoglycerate mutase	O70250	29	4			
	γC-crystallin	Q61597	21	1			
	γB-crystallin	Q6PHP7	21	1			
5468	Phosphoglycerate mutase	O70250	29	2	−1.03	−2.4	−2.52
5492	βB1-crystallin	Q9WVJ5	28	1	1.17	−2.95	−3.72
	Riken cDNA2210010C04 isoformCRA-b	Q9CPN9	26	1			
5960	Putative uncharacterized protein	Q8C2C1	20	1	1.3	−2.52	−3.52
	γB-crystallin	Q6PHP7	21	1			
	Riken cDNA2210010C04 isoformCRA-b	Q9CPN9	26	1			
6005	γC-crystallin	Q61597	21	1	1.54	−1.81	−2.99
6056	αB-crystallin	P23927	20	25	−1.17	9.23	10.02
	βB2-crystallin	P62696	23	10			
	βA3/A1-crystallin	Q9QXC6	25	9			
	αA-crystallin	Q569M7	20	7			
	βS-crystallin	O35486	21	4			
	γD-crystallin	Q6PGI0	21	4			
	βB3-crystallin	Q9JJU9	24	4			
	γB-crystallin	Q6PHP7	21	2			
	βA2-crystallin	Q9JJV1	22	2			
6415	αA-crystallin	Q569M7	20	7	3.28	10.01	2.83
6449	αA-crystallin	Q569M7	20	53	2	12.67	5.87

Table 4. Cont.

Spot number	Protein	UNIPROT accession number	MW (kDa)	Number of assigned spectra	WT1 vs. WT2	WT1 vs. heterozygous	WT2 vs. heterozygous
6848	Grifin	Q9D1U0	16	5	−1.04	2.42	2.33
	αA-crystallin	Q569M7	20	5			
6920	αA-crystallin	Q569M7	20	4	−1.55	1.89	2.71
7257	αA-crystallin	Q569M7	20	6	−1.13	2.7	2.84
7451	αB-crystallin	P23927	20	6	1.13	5.61	4.59
	αA-crystallin	Q569M7	20	5			
	βB3-crystallin	Q9JJU9	24	2			
7739	αA-crystallin	Q569M7	20	5	−1.11	2.39	2.47
	Riken cDNA2210010C04 isoform CRA-b	Q9CPN9	26	1			
6061	γC-crystallin	Q61597	21	2	−1.04	−2.63	−2.73
	αA-crystallin	Q569M7	20	2			
	γB-crystallin	Q6PHP7	21	2			
	γD-crystallin	Q6PGI0	21	1			
	γA-crystallin	P04345	21	1			

Spot number	Protein	Uniprot accession number	MW (kDa)	Number of assigned spectra	Fold change		
					WT1 vs WT2	WT1 vs Homozygous	WT2 vs Homozygous
5938	αB-crystallin	P23927	20	16	1.42	−1.85	−2.79
	βS-crystallin	O35486	21	10			
	γB-crystallin	Q6PHP7	21	7			
	βA3/A1-crystallin	Q9QXC6	25	6			
	γD-crystallin	Q6PGI0	21	5			
	αA-crystallin	Q569M7	20	5			
	γC-crystallin	Q61597	21	3			
	γF-crystallin	Q9CXV3	21	3			
	βB3-crystallin	Q9JJU9	24	2			
5961	αB-crystallin	P23927	20	40	1.12	8.62	7.25
	βS-crystallin	O35486	21	8			
	γD-crystallin	Q6PGI0	21	7			
	βA3/A1-crystallin	Q9QXC6	25	6			
	γB-crystallin	Q6PHP7	21	5			
	αA-crystallin	Q569M7	20	4			

Table 4. Cont.

Spot number	Protein	Uniprot accession number	MW (kDa)	Number of assigned spectra	Fold change		
					WT1 vs WT2	WT1 vs Homozygous	WT2 vs Homozygous
	γN-crystallin	Q8VHL5	21	2			
	γF-crystallin	Q9CXV3	21	2			
	Fatty acid binding protein	Q05816	15	2			
	γC-crystallin	Q61597	21	1			
5963	αB-crystallin	P23927	20	43	1.01	−5.61	−6.05
	γD-crystallin	Q6PGI0	21	10			
	βS-crystallin	O35486	21	9			
	γB-crystallin	Q6PHP7	21	7			
	γF-crystallin	Q9CXV3	21	3			
	γC-crystallin	Q61597	21	1			
	βA3/A1-crystallin	Q9QXC6	25	1			
6120	αB-crystallin	P23927	20	9	2.14	4.31	1.89
	αA-crystallin	Q569M7	20	5			
	γB-crystallin	Q6PHP7	21	1			
7164	αB-crystallin	P23927	20	3	−1.11	1.98	2.07
	αA-crystallin	Q569M7	20	1			

WT, wild-type.
doi:10.1371/journal.pone.0095507.t004

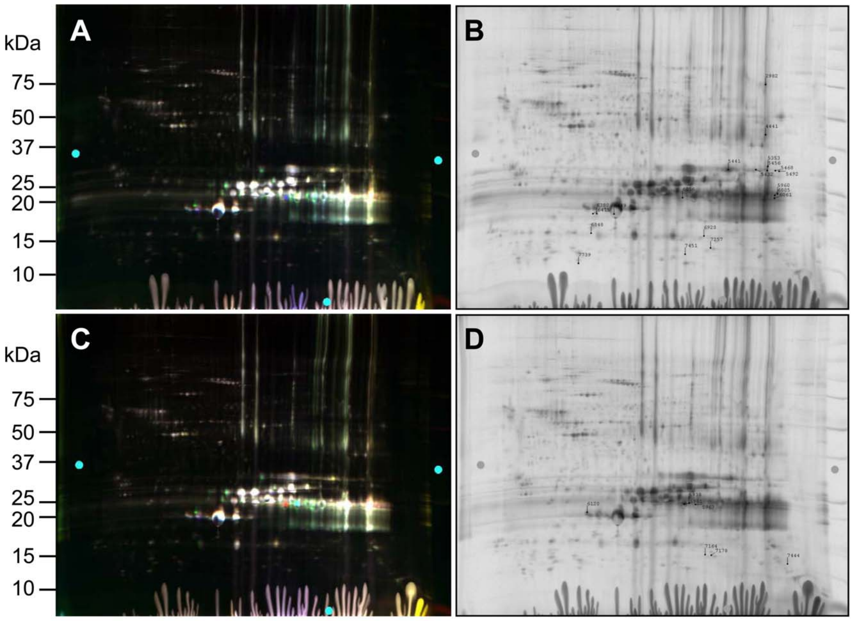

Figure 6. 2D-DIGE analysis of proteomic changes in whole lenses of 14-day-old mice induced by knock-in of the αB-R120G mutation. (A) 2D gel of lens proteins labeled with cyanine dyes derived from WT1 proteins labeled with Cy5, WT2 proteins labeled with Cy3, and αB-R120G heterozygous lens proteins labeled with Cy2. (B) 2D gel of lens proteins labeled with cyanine dyes derived from WT1 proteins labeled with Cy2, WT2 proteins labeled with Cy3, and αB-R120G homozygous proteins labeled with Cy5. (C, D) Protein spots that were selected for analysis from the gel shown in (A) and (B) are shown in (C) and (D), respectively. Proteins were identified by tandem mass spectrometry and Mascot searches of spots that were selected from analysis of the gels. Quantitative image analysis and mass spectrometry data for the identified proteins from these gels are listed in Table 4.
doi:10.1371/journal.pone.0095507.g006

amounts of certain proteins were greater in the complete absence of a WT αA-crystallin gene (homozygous mutant) than with only one copy of the WT gene (heterozygous mutant). Examples are shown in Tables 1-3 for the αA-R49C protein. The effect of developmental age was investigated using 2- and 14-day-old R49C mutant lenses (Fig. S2 in File S1 and Table S1). The increased abundance of several proteins and the degradation of αA-crystallin previously observed in 2-day-old homozygous mutant lenses were confirmed at 14 days.

An important conclusion of the present study is that the αB-R120G mutation causes specific *in vivo* changes in protein abundance. Protein changes in the αB-R120G lenses were distinctly different from those in αA-R49C mutant lenses. The main changes in the αB-R120G mutant lens included altered abundance of β- and γ-crystallins, increased degradation of αA-, αB-, and γ-crystallins, and degradation of phosphoglycerate mutase, a glycolytic enzyme that is very important in metabolism but has not been studied in the lens in detail [46–49]. There was also a 12-fold increase in the amount of αA-crystallin associated with grifin in these lenses.

Our studies demonstrated that 2-week-old αA-R49C homozygous lenses contained a high abundance of low molecular weight proteins (<14 kDa) indicating that the absence of WT αA-crystallin leads to protein instability, greater susceptibility to proteolysis, and protein degradation. This occurred as a primary event at an early postnatal stage. Previous studies have identified lens protein truncation with age in human lenses [50,51]. In future work, we intend to identify the common structural features that make the proteins more labile to proteolysis, which will provide critical information needed to develop a model of *in vivo* cataract formation. Our previous studies involving molecular weight measurements of the αA-R49C homozygous lenses by light scattering also demonstrated an increase in low molecular weight proteins (~15 kDa) in these lenses [10]. We first examined the presence of low molecular weight proteins in the homozygous lenses, and then compared WT, heterozygous, and homozygous lenses. We subsequently identified the low molecular weight proteins as αA-crystallin associated with other crystallins, gelsolin and degraded ceruloplasmin, that were absent from WT mouse lenses but abundant in 2-week-old αA-R49C homozygous lenses (Table 3).

αA- and αB-crystallins were degraded in both αA-R49C and αB-R120G mutant lenses at a young age, suggesting that the mutations make these proteins less stable. Decreased stability was associated with increased crosslinking of αA-crystallin, as shown by the 15-fold increase in crosslinking of αA-crystallin to form a higher molecular weight form of approximately 40 kDa that corresponded to a crosslinked dimer. We detected increased crosslinking of αA-crystallin very early, even in lenses of 2-day-old postnatal αA-R49C heterozygous mice. Previous studies have shown that increased crosslinking can reduce the chaperone activity of α-crystallin [52]. We previously used immunoblot analysis to show an increase in the amount of water-insoluble αB-crystallin in 6-week-old αB-R120G mutant lenses [17]. We now demonstrate the presence of high molecular weight αB-crystallin in postnatal αB-R120G heterozygous and homozygous lenses, indicating that they appear early during postnatal development and consistent with their important role in opacification of αB-R120G heterozygous and homozygous lenses.

In previous studies we investigated the effect of αA/B double knock-out. The expression of βB2-crystallin increased 40-fold in 6-week-old αA/B DKO lens epithelial cells; however, the upregulation of βB2-crystallin protein was not observed in 2-day-old DKO lenses, indicating that this was not a physiological stress-induced

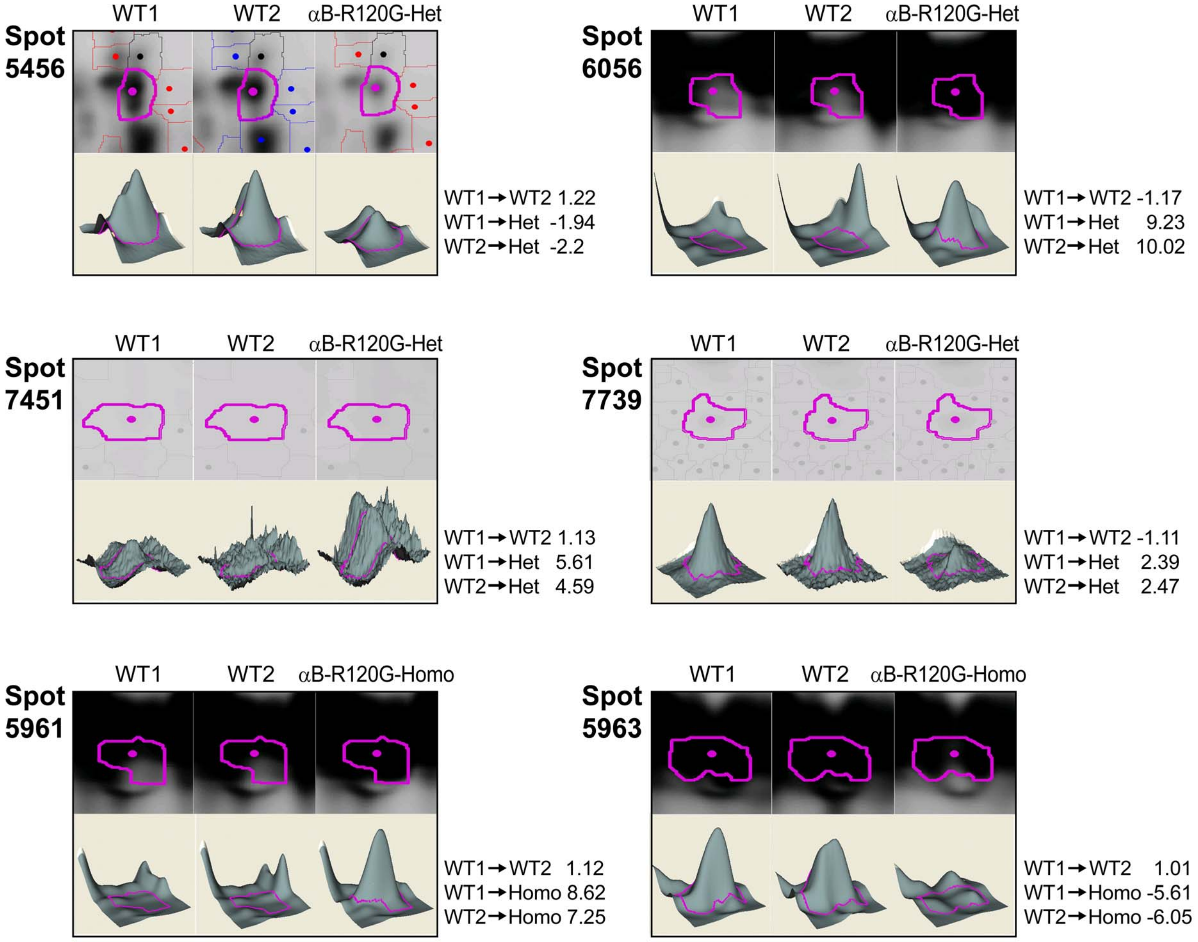

Figure 7. Quantitative analysis of the changes in abundance of proteins in postnatal 14-day-old lens from WT and αB-R120G knock-in mice by mass spectrometry. The 3D data sets for representative proteins in two WT (WT1 and WT2) and one αB-R120G mutant sample are shown. (A) WT1 and WT2 proteins were labeled with Cy3 and Cy5 dyes, respectively, and αB-R120G heterozygous mutant lenses with Cy2. (B) WT1 and WT2 proteins were labeled with Cy2 and Cy3 dyes, respectively, and αB-R120G homozygous mutant lenses with Cy5. Fold changes between each sample are indicated on the right. See Table 4 for the identity of proteins present in each protein spot.
doi:10.1371/journal.pone.0095507.g007

effect but probably developmental. Surprisingly, in 6-week-old DKO mouse lenses we did not observe an increase of lower molecular weight (<14 kDa) proteins as seen in the knock-in lenses. This was the major difference between αA/B DKO lenses and αA-R49C homozygous lenses although there were other distinct differences between the proteins altered in knock-out versus knock-in αA-R49C mutant and αB-R120G mutant lenses. For example, the following effects were observed only in knock-in mutant lenses: increased abundance of creatine kinase B associated with αA-crystallin (only in αA-R49C mutant lenses); decreased abundance of phosphoglycerate mutase; changes in grifin associated with αA-crystallin; association of chaperones of the HSP70 and TCP-1 families with αA-crystallin (only in the αA-R49C mutant lenses); decreased abundance of in γ-crystallins; increased abundance of the apoptotic protein annexin. In contrast, degradation of titin, β1-catenin, and a decrease in serine threonine protein kinase were observed only in αA/αB DKO lenses. However, common features in our analyses of αA/αB-knock-out lenses and the αA-R49C and αB-R120G mutant knock-in lenses included changes in histones, hemoglobin, glutamate dehydrogenase, GST-μ, and βB1-crystallin. An increase in βB1-crystallin crosslinking and degradation was observed in the knock-in mutant lenses, but only its crosslinking increased in the knock-out lenses. Crosslinking of vimentin, tubulin, and actin increased and their abundance decreased in both knock-out and knock-in lenses. These differences in protein abundance and degradation among the three model systems indicate that specific cellular conditions dictate the substrates for α-crystallins during the early stages of lens development. This reveals variable substrate recognition by α-crystallins, which when fully understood may provide insights into how to limit the damage resulting from protein unfolding in cataracts and could implicate use of the aggregation-preventing properties of α-crystallins to control damage due to stress and disease.

It has been proposed that a combination of interaction sites could be key in substrate recognition by αA-crystallin [53]. The interaction of α-crystallins with substrate proteins is non-covalent in nature, and hydrophobic interactions need only a subtle change on the protein surface of the target proteins. Hydrophobic interactions are probably more common than previously believed because proteins are dynamic systems. A very small area might become exposed and bind to a hydrophobic surface on the chaperone protein even though the particle size may not change sufficiently to cause light scattering. Moreover, changes in the pI of proteins can occur without a stability change. Surface anisotropy can change many times in response to unidentified factors in the

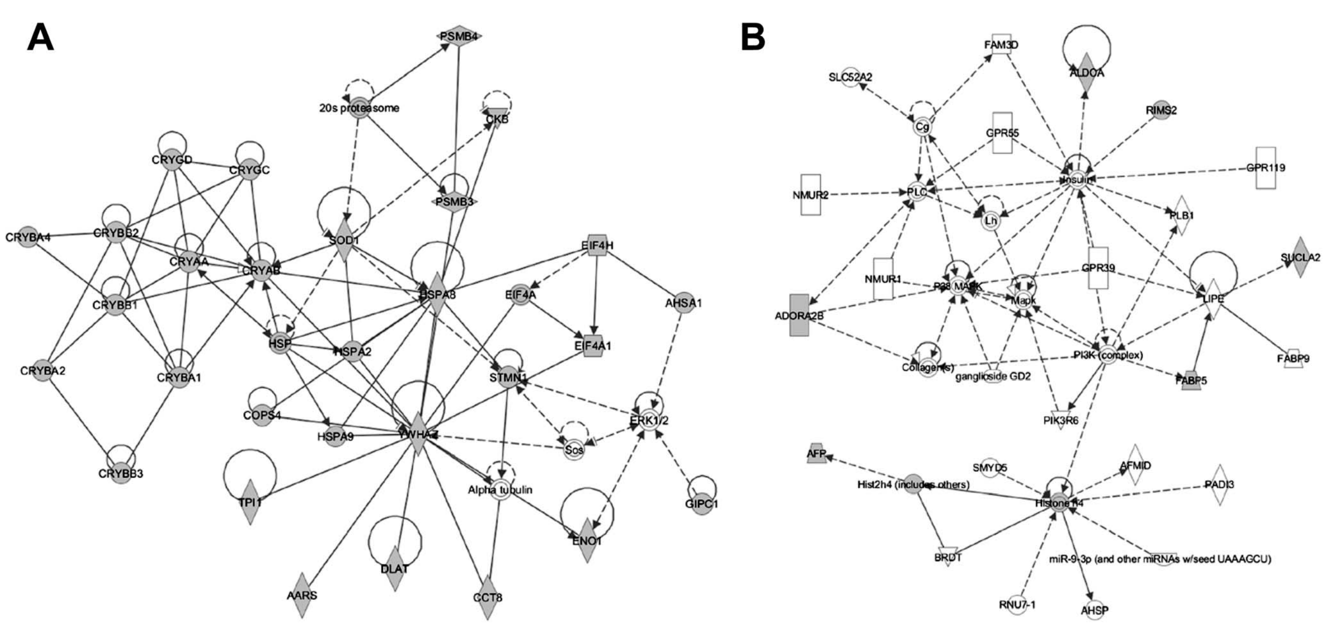

Figure 8. Ingenuity Pathways analysis of lens proteins identified in αA-R49C knock-in mutant lenses. Analysis of altered protein networks by Ingenuity Pathway software. Biological networks and pathways generated from input data (Wild-type vs. αA-R49C, Tables 1-3 and Table S1) indicate proteins with altered abundance in gray. (A) A network with HSPA8 at the hub. (B) A second network highlights Histone H4 at the hub of the protein connectivity map. Additional networks are shown in Fig. S3 in File S1.
doi:10.1371/journal.pone.0095507.g008

environment of cells. There is no change in protein size in many hereditary cataracts caused by γ-crystallin mutations, instead the cataract is formed by increased electrostatic interaction between the positively charged E107A γD-crystallin and the negatively charged α-crystallins, which increases the amount of light scattering [54,55]. This may also occur in αA-R49C and αB-R120G mutant proteins in which the negative charge on arginine is lost when it is replaced by cysteine or glycine, respectively, and the proteins have a more acidic pI, resulting in an increase in light scattering. Thurston *et al.* showed that the strength of the interaction between native γ- and α-crystallins is essentially optimal for lens transparency, and that a small increase in this interaction can increase light scattering and lead to cataract [56,57]. Further studies are needed to elucidate the hierarchy in the interaction of αA- and αB-crystallins with different proteins and the interactive sequences involved.

In summary, our studies demonstrate that characterization of changes in protein abundance in postnatal lenses is an effective way to identify *in vivo* substrates of αA- and αB-crystallins. Proteins that showed the greatest change in abundance at an early age are very likely to be *in vivo* substrates of the α-crystallins. Further quantitative studies are required to define the relationship(s) between binding of αA- and αB-crystallins and polymerization and subcellular distribution of the substrates identified in this study. This will provide new information into protein abundance changes that may occur in cataracts, even before the opacification process becomes obvious. Our approach could therefore characterize the *in vivo* state at the beginning of cataract development in the mouse lens, providing information necessary to develop interventional strategies to prevent future lens opacities.

Materials and Methods

Animals and Lenses

αA-R49C knock-in mice and αB-R120G knock-in mice were generated by stem cell-based techniques as described previously [17]. Mice were converted to the C57 background using speed congenics. Wild type (WT), heterozygous mutant, and homozygous mutant mice used in this study were genotyped by PCR-based methods. All procedures involving mice were performed by trained veterinary staff at the Mouse Genetics Core at Washington University. All protocols and animal procedures were approved by the Washington University Animal Studies Committee (protocol number 20110258). Lenses from two different age groups of αA-R49C knock-in mice (2-day old and 2-week-old) were analyzed by mass spectrometry (2-4 mice in each replicate set of WT1, WT2, and αA-R49C heterozygous mice and WT1, WT2, and αA-R49C homozygous mice). WT and αA-R49C knock-in mutant lenses were subjected to two-dimensional difference gel electrophoresis (2D-DIGE). Lenses from 2-week-old αB-R120G heterozygous and homozygous mice were also analyzed by 2D-DIGE.

Mass Spectrometric Analysis

Lenses were dissected and placed in lysis buffer containing 30 mM Tris-HCl (BioRad, Hercules, CA), 2 M thiourea (Sigma-Aldrich, St. Louis, MO), 7 M urea (BioRad), 4% CHAPS (BioRad), and 1× complete protease inhibitor cocktail tablets (Roche, Indianapolis, IN), pH 8.5. Lens proteins (50 µg) were labeled with 400 pmol Cy2, Cy3, or Cy5. Pools were prepared by mixing equal quantities of protein from each sample after dye labeling [58]. 2D-DIGE was performed at the Proteomics Core Laboratory according to published methods [59]. Briefly, samples were equilibrated onto immobilized pH gradient strips at 100 V and subjected to isoelectric focusing using a maximum of 10,000 focusing volts (PROTEAN IEF cell: BioRad). After focusing, proteins were reduced with Tris(2-carboxyethyl) phosphine hydrochloride (TCEP, 10 mM) and alkylated with iodoacetamide (20 mM). The strip was then layered on a 10-20% polyacrylamide gel, and proteins were separated by SDS-PAGE. Samples were imaged with a Typhoon 9400 Imager (GE Healthcare, Piscataway, New Jersey) using specific excitation and emission wave-

lengths for Cy2 (488 and 522 nm), Cy3 (520 and 580 nm), and Cy5 (620 and 670 nm). Control and experimental samples were labeled with blue or red fluorescent dyes and run on the same 2D gel [60,61]. Image analysis was performed to assess differences between WT and homozygous/heterozygous mutant lenses. Individual protein spots that showed differential intensities were excised from the gel and analyzed by mass spectroscopy. Fold changes represented proteins with increased (positive numbers) or decreased (negative numbers) expression in mutant versus WT samples.

Single or multi-gel analyses were used to determine changes in protein abundance between WT and knock-in mouse lenses. Single gel analysis was performed to compare the following conditions: WT and αA-R49C heterozygous and homozygous mutant lenses (Tables 1, 3, Figs. 1 and 2, Table S1), and WT and αB-R120G heterozygous and homozygous mutant lenses (Table 4, Figs. 6 and 7). In addition, multi-gel analysis was performed with a pooled internal standard. This approach was used to compare 2-day-old WT, αA-R49C heterozygous, and homozygous mutant lenses (Table 2 and Figs. 3 and 4). Multi-gel comparisons were performed using different combinations of sample sets. The WT sample was labeled with Cy2 and mutant samples were labeled with Cy5. A pool of all samples was labeled with Cy3 and served as a standard that was common to each gel. The pooled standard, the control, and one test sample were combined and run on each gel. Images were generated and compared within each 2D gel using DeCyder v.6.5 image analysis software (GE Healthcare). Differential in-gel analysis (DIA) was used to normalize and compare quantitative differences between images from each gel. Image analysis using DeCyder software generates a relative value for the abundance of the spot in different samples, but there is no mechanism to determine the statistical significance of the differences. We therefore performed analysis of combined biological replicates for the different genotypes. In addition, we used Biological Variation Analysis (BVA) for the 2-day-old αA-R49C knock-in mouse lenses to obtain statistical significance as described below [59].

Analysis of Pool-Based Data

Pool-based studies involved a pool of proteins from all samples in the experiment, providing a common comparator for each sample. Because the pool is identical on each gel, the fold change "difference" for a spot in the pool image is 1.0 (representing no change) when comparing pool images from any two gels. This designation allowed us to compare protein amounts for spots of WT or αA-R49C heterozygous lens samples to the pool on the same gel to determine relative amounts of protein. Although WT and mutant samples were resolved on different gels, their fold changes were determined in comparison to the pooled sample, which was also run on each gel. Because the pool from one gel is identical to the pool from another, the WT and mutant fold change values could be directly compared. Pairwise analysis of proteins across different physical gels was performed using the BVA module to quantify relative differences between the samples [59]. BVA compares the quantitative value of the spot as it is represented among different samples. BVA data generates *t*-test and assigns p value to identify statistical significance. $p < 0.05$ denotes statistical significance (Table 2).

Database Searching

The mass spectra were acquired using nano-LC-MS as previously described [62]. All tandem mass spectrometry samples were analyzed using Mascot (Matrix Science, London, UK; version 2.1.1.0) as previously described [23]. Mascot was set to search the Uniprot mouse database (downloaded 12/28/2010, 135387 entries) using trypsin as the digestion enzyme, with a fragment ion mass tolerance of 0.80 Da and a parent ion tolerance of 50 ppm for data from the LTQ FT mass spectrometer. The QSTAR data were searched using a parent and fragment tolerance of 0.1 Da. The iodoacetamide derivative of cysteine was specified in Mascot as a fixed modification and methionine as a variable modification. Scaffold software (v. 3.6.1) was used to display proteomic data. Additional data processing details have been previously described [59].

Criteria for Protein Identification

Scaffold (version Scaffold_3_01_00, Proteome Software Inc., Portland, OR) was used to qualify MS/MS-based peptide and protein identifications [63]. Protein identification was accepted if identity could be established at >95.0% probability and involved at least one identified peptide. Protein probabilities were assigned using the Protein Prophet algorithm ([64] AI et al 2003). Proteins that contained similar peptides but were not differentiated based on MS/MS analysis alone were grouped to satisfy the principles of parsimony. Mass spectra for all the proteins identified in this study are shown in Table S4.

Knowledge-based Network Analysis

After false positive analysis (Protein Prophet) and removal of contaminants (e.g., keratins), proteins listed in Tables 1-4 and S1 (identified by UNIPROT accession numbers) were entered into Ingenuity Pathways (www.ingenuity.com) (IPA, version 8.8, Redwood City, CA) as a *.xls file. The software mapped 99 of 118 Gi numbers, corresponding to 99 gene symbols. Duplicate names corresponding to the same gene were eliminated. Ingenuity was set to generate up to 25 networks containing up to 35 members each, with no additional restrictions. Biological networks and pathways were generated from the input data ('focus genes') and gene objects in the Ingenuity Pathways Knowledge Base (IPKB). Interaction networks generated using this method showed proteins present in our samples as shaded in grey and other interacting proteins not identified from these gels as unshaded.

Supporting Information

Table S1 Analysis of proteins that showed differences in abundance between 2-day-old WT, 14-day-old WT and 2-day-old αA-R49C homozygous mouse lenses. WT, Wild-type.
(DOC)

Table S2 Ingenuity Pathway Analysis (IPA) molecules table for proteins affected by αA-crystallin R49C mutation in the mouse lens.
(XLS)

Table S3 Ingenuity Pathway Analysis (IPA) molecules table for proteins affected by αB-crystallin R120G mutation in the mouse lens.
(XLS)

Table S4 Mass spectrometry and database search results for proteins identified in this study.
(XLSX)

File S1 Supplementary figures. **Figure S1**, 2D-DIGE analysis of proteomic changes in whole lenses of 2-day-old mice with knock-in of the αA-R49C mutation. Protein spots that were picked for analysis from the 2D gels of WT and αA-R49C heterozygous (A) and WT and αA-R49C homozygous lenses (B-D)

shown in Figure 1. Quantitative image analysis and mass spectrometry data for identified proteins from these gels are listed in Table 1. **Figure S2**, 2D-DIGE analysis of proteomic changes in whole lenses of 2-day-old and 14-day-old mice induced by knock-in of the αA-R49C mutation. (A) A 2D gel of lens proteins labeled with cyanine dyes derived from 2-day-old WT proteins labeled with Cy3, 14-day-old WT proteins labeled with Cy5, and αA-R49C homozygous lens proteins labeled with Cy2. (B, C) Protein spots that were selected for analysis from the gel shown in (A). Proteins were identified by tandem mass spectrometry and Mascot searches of spots that were selected from the gels. Quantitative image analysis and mass spectrometry data for the identified proteins from these gels are listed in Table S1. **Figure S3**, Protein connectivity networks identified by Ingenuity Pathway analysis of lens proteins in αA-R49C knock-in mutant lenses. Analysis of altered protein networks by Ingenuity Pathway software. Biological networks and pathways generated from input data (Wild type vs. αA-R49C, Tables 1-3 and S1) indicate proteins with changed abundance in gray. (A) A network with GAPDH at the hub. (B) A second network with F-actin at the hub. (C) A third network highlights NPM1 at the hub of the protein connectivity map. (D) A fourth network with TGFB1 at the hub. (E) A fifth network indicates the interaction between grifin and IKZF1. (F) A sixth network shows Gm5409 at the hub. Note that two additional networks are shown in Figure 8. **Figure S4**, Networks revealed by Ingenuity Pathway analysis of lens proteins that changed in amount in WT vs. αB-R120G knock-in lenses. Biological networks and pathways generated from input data (Wild type vs. αB-R120G, Table 4) indicate proteins with changed abundance in gray. (A) A network with MAF at the hub. (B) A second network with UBC at the hub. (C) A third network shows the interactions between grifin and IKZF1. (D) A fourth network highlights CTRB2 at the hub of the protein connectivity map.
(DOC)

Author Contributions

Conceived and designed the experiments: UPA JPM RRT. Performed the experiments: UPA JPM. Analyzed the data: UPA JPM RRT. Contributed reagents/materials/analysis tools: UPA JPM RRT. Wrote the paper: UPA JPM RRT.

References

1. Andley UP (2007) Crystallins in the eye: Function and pathology. Prog Retin Eye Res 26: 78–98.
2. Andley UP (2008) The lens epithelium: focus on the expression and function of the alpha-crystallin chaperones. Int J Biochem Cell Biol 40: 317–323.
3. Bhat S (2002) The ocular lens epithelium. Bioscience Reports 21: 537–563.
4. Wang X, Garcia CM, Shui YB, Beebe DC (2004) Expression and regulation of alpha-, beta-, and gamma-crystallins in mammalian lens epithelial cells. Invest Ophthalmol Vis Sci 45: 3608–3619.
5. Robinson ML, Overbeek PA (1996) Differential expression of alpha A- and alpha B-crystallin during murine ocular development. Invest Ophthalmol Vis Sci 37: 2276–2284.
6. Bloemendal H, de Jong W, Jaenicke R, Lubsen NH, Slingsby C, et al. (2004) Ageing and vision: structure, stability and function of lens crystallins. Prog Biophys Mol Biol 86: 407–485.
7. Horwitz J (1992) Alpha-crystallin can function as a molecular chaperone. Proc Natl Acad Sci U S A 89: 10449–10453.
8. Vicart P, Caron A, Guicheney P, Li Z, Prevost MC, et al. (1998) A missense mutation in the alphaB-crystallin chaperone gene causes a desmin-related myopathy. Nat Genet 20: 92–95.
9. Andley UP (2006) Crystallins and hereditary cataracts: molecular mechanisms and potential for therapy. Expert Rev Mol Med 8: 1–19.
10. Andley UP, Hamilton PD, Ravi N (2008) Mechanism of insolubilization by a single-point mutation in alphaA-crystallin linked with hereditary human cataracts. Biochemistry 47: 9697–9706.
11. Pande A, Zhang, J, Shekhtman, A, Pande, J. A high resolution NMR study of human gammaD crystallin and its cataract-linked P23T mutant: Small conformational changes in P23T explain its low and retrograde solubility.; 2008. pp. 284.
12. Xia CH, Liu H, Chang B, Cheng C, Cheung D, et al. (2006) Arginine 54 and Tyrosine 118 residues of {alpha}A-crystallin are crucial for lens formation and transparency. Invest Ophthalmol Vis Sci 47: 3004–3010.
13. Hsu CD, Kymes S, Petrash JM (2006) A transgenic mouse model for human autosomal dominant cataract. Invest Ophthalmol Vis Sci 47: 2036–2044.
14. Huang Q, Ding L, Phan KB, Cheng C, Xia CH, et al. (2009) Mechanism of cataract formation in alphaA-crystallin Y118D mutation. Invest Ophthalmol Vis Sci 50: 2919–2926.
15. Mackay DS, Andley UP, Shiels A (2003) Cell death triggered by a novel mutation in the alphaA-crystallin gene underlies autosomal dominant cataract linked to chromosome 21q. Eur J Hum Genet 11: 784–793.
16. Koteiche HA, McHaourab HS (1999) Folding pattern of the alpha-crystallin domain in alphaA-crystallin determined by site-directed spin labeling. J Mol Biol 294: 561–577.
17. Andley UP, Hamilton PD, Ravi N, Weihl CC (2011) A knock-in mouse model for the R120G mutation of alphaB-crystallin recapitulates human hereditary myopathy and cataracts. PLoS One 6: e17671.
18. Liu Y, Zhang X, Luo L, Wu M, Zeng R, et al. (2006) A Novel {alpha}B-Crystallin Mutation Associated with Autosomal Dominant Congenital Lamellar Cataract. Invest Ophthalmol Vis Sci 47: 1069–1075.
19. Ghosh JG, Estrada MR, Clark JI (2005) Interactive domains for chaperone activity in the small heat shock protein, human alphaB crystallin. Biochemistry 44: 14854–14869.
20. Ghosh JG, Shenoy AK, Jr., Clark JI (2007) Interactions between important regulatory proteins and human alphaB crystallin. Biochemistry 46: 6308–6317.
21. Bates GP (2006) BIOMEDICINE: One Misfolded Protein Allows Others to Sneak By. Science 311: 1385–1386.
22. Gidalevitz T, Ben-Zvi A, Ho KH, Brignull HR, Morimoto RI (2006) Progressive disruption of cellular protein folding in models of polyglutamine diseases. Science 311: 1471–1474.
23. Andley UP, Malone JP, Hamilton PD, Ravi N, Townsend RR (2013) Comparative proteomic analysis identifies age-dependent increases in the abundance of specific proteins after deletion of the small heat shock proteins alphaA- and alphaB-crystallin. Biochemistry 52: 2933–2948.
24. Sharma K, Vabulas RM, Macek B, Pinkert S, Cox J, et al. (2012) Quantitative proteomics reveals that Hsp90 inhibition preferentially targets kinases and the DNA damage response. Mol Cell Proteomics 11: M111 014654.
25. Koteiche HA, McHaourab HS (2006) Mechanism of a hereditary cataract phenotype. Mutations in alphaA-crystallin activate substrate binding. J Biol Chem 281: 14273–14279.
26. Toyama BH, Hetzer MW (2013) Protein homeostasis: live long, won't prosper. Nat Rev Mol Cell Biol 14: 55–61.
27. Xi JH, Bai F, Gross J, Townsend RR, Menko AS, et al. (2008) Mechanism of small heat shock protein function in vivo: a knock-in mouse model demonstrates that the R49C mutation in alpha A-crystallin enhances protein insolubility and cell death. J Biol Chem 283: 5801–5814.
28. Andley UP, Patel HC, Xi JH (2002) The R116C mutation in alpha A-crystallin diminishes its protective ability against stress-induced lens epithelial cell apoptosis. J Biol Chem 277: 10178–10186.
29. Sultana R, Boyd-Kimball D, Poon HF, Cai J, Pierce WM, et al. (2006) Redox proteomics identification of oxidized proteins in Alzheimer's disease hippocampus and cerebellum: an approach to understand pathological and biochemical alterations in AD. Neurobiol Aging 27: 1564–1576.
30. Sabah JR, Davidson H, McConkey EN, Takemoto L (2004) In vivo passage of albumin from the aqueous humor into the lens. Mol Vis 10: 254–259.
31. Sabah J, McConkey E, Welti R, Albin K, Takemoto LJ (2005) Role of albumin as a fatty acid carrier for biosynthesis of lens lipids. Exp Eye Res 80: 31–36.
32. Sabah JR, Schultz BD, Brown ZW, Nguyen AT, Reddan J, et al. (2007) Transcytotic passage of albumin through lens epithelial cells. Invest Ophthalmol Vis Sci 48: 1237–1244.
33. Delcourt C, Dupuy AM, Carriere I, Lacroux A, Cristol JP, et al. (2005) Albumin and transthyretin as risk factors for cataract: the POLA study. Arch Ophthalmol 123: 225–232.
34. Gokhin DS, Nowak RB, Kim NE, Arnett EE, Chen AC, et al. (2012) Tmod1 and CP49 synergize to control the fiber cell geometry, transparency, and mechanical stiffness of the mouse lens. PLoS One 7: e48734.
35. Andley UP, Reilly MA (2010) In vivo lens deficiency of the R49C alphaA-crystallin mutant. Experimental Eye Research 90: 699–702.
36. Barton KA, Hsu CD, Petrash JM (2009) Interactions between small heat shock protein alpha-crystallin and galectin-related interfiber protein (GRIFIN) in the ocular lens. Biochemistry 48: 3956–3966.
37. Clark TJ, Houck SA, Clark JI (2012) Hemoglobin interactions with alphaB crystallin: a direct test of sensitivity to protein instability. PLoS One 7: e40486.
38. Srivastava OP, Kirk MC, Srivastava K (2004) Characterization of covalent multimers of crystallins in aging human lenses. J Biol Chem 279: 10901–10909.
39. Wang Z, Lyons B, Truscott RJ, Schey KL (2014) Human protein aging: modification and crosslinking through dehydroalanine and dehydrobutyrine intermediates. Aging Cell 13: 226–234.

40. Lampi KJ, Amyx KK, Ahmann P, Steel EA (2006) Deamidation in human lens betaB2–crystallin destabilizes the dimer. Biochemistry 45: 3146–3153.
41. Michiel M, Duprat E, Skouri-Panet F, Lampi JA, Tardieu A, et al. (2010) Aggregation of deamidated human betaB2-crystallin and incomplete rescue by alpha-crystallin chaperone. Exp Eye Res 90: 688–698.
42. Putilina T, Skouri-Panet F, Prat K, Lubsen NH, Tardieu A (2003) Subunit exchange demonstrates a differential chaperone activity of calf alpha-crystallin toward beta LOW- and individual gamma-crystallins. J Biol Chem 278: 13747–13756.
43. Ajaz MS, Ma Z, Smith DL, Smith JB (1997) Size of human lens beta-crystallin aggregates are distinguished by N-terminal truncation of betaB1. J Biol Chem 272: 11250–11255.
44. McHaourab HS, Kumar MS, Koteiche HA (2007) Specificity of alphaA-crystallin binding to destabilized mutants of betaB1-crystallin. FEBS Lett 581: 1939–1943.
45. Dolinska MB, Sergeev YV, Chan MP, Palmer I, Wingfield PT (2009) N-terminal extension of beta B1-crystallin: identification of a critical region that modulates protein interaction with beta A3-crystallin. Biochemistry 48: 9684–9695.
46. Ohrloff C, Berdjis H, Hockwin O, Bours J (1983) Age-related changes of glyceraldehyde-3-phosphate-dehydrogenase (E.C. 1.2.1.12), 3-phosphoglycera-tekinase (E.C. 2.7.2.3), phosphoglyceratemutase (E.C. 2.7.5.3), and enolase (E.C. 4.2.1.11) in bovine lenses. Ophthalmic Res 15: 293–299.
47. Takata T, Shimo-Oka T, Miki K, Fujii N (2005) Characterization of new D-beta-aspartate-containing proteins in a lens-derived cell line. Biochem Biophys Res Commun 334: 1022–1031.
48. Hoehenwarter W, Kumar NM, Wacker M, Zimny-Arndt U, Klose J, et al. (2005) Eye lens proteomics: from global approach to detailed information about phakinin and gamma E and F crystallin genes. Proteomics 5: 245–257.
49. St-Amand J, Okamura K, Matsumoto K, Shimizu S, Sogawa Y (2001) Characterization of control and immobilized skeletal muscle: an overview from genetic engineering. FASEB J 15: 684–692.
50. Grey AC, Schey KL (2009) Age-related changes in the spatial distribution of human lens alpha-crystallin products by MALDI imaging mass spectrometry. Invest Ophthalmol Vis Sci 50: 4319–4329.
51. Lampi KJ, Ma Z, Hanson SR, Azuma M, Shih M, et al. (1998) Age-related changes in human lens crystallins identified by two-dimensional electrophoresis and mass spectrometry. Exp Eye Res 67: 31–43.
52. Bhattacharyya J, Shipova EV, Santhoshkumar P, Sharma KK, Ortwerth BJ (2007) Effect of a single AGE modification on the structure and chaperone activity of human alphaB-crystallin. Biochemistry 46: 14682–14692.
53. Ghosh JG, Houck SA, Clark JI (2008) Interactive sequences in the molecular chaperone, human alphaB crystallin modulate the fibrillation of amyloidogenic proteins. Int J Biochem Cell Biol 40: 954–967.
54. Banerjee PR, Pande A, Patrosz J, Thurston GM, Pande J (2011) Cataract-associated mutant E107A of human gammaD-crystallin shows increased attraction to alpha-crystallin and enhanced light scattering. Proc Natl Acad Sci U S A 108: 574–579.
55. Asherie N (2011) Blind attraction: the mechanism of an inherited congenital cataract. Proc Natl Acad Sci U S A 108: 437–438.
56. Dorsaz N, Thurston GM, Stradner A, Schurtenberger P, Foffi G (2009) Colloidal characterization and thermodynamic stability of binary eye lens protein mixtures. J Phys Chem B 113: 1693–1709.
57. Stradner A, Foffi G, Dorsaz N, Thurston G, Schurtenberger P (2007) New insight into cataract formation: enhanced stability through mutual attraction. Phys Rev Lett 99: 198103.
58. Andley UP, Malone JP, Townsend RR (2011) Inhibition of Lens Photodamage by UV-Absorbing Contact Lenses. Investigative Ophthalmology and Visual Science 52: 8330–8341.
59. Mendelsohn BA, Malone JP, Townsend RR, Gitlin JD (2009) Proteomic analysis of anoxia tolerance in the developing zebrafish embryo. Comp Biochem Physiol Part D Genomics Proteomics 4: 21–31.
60. Hu Y, Malone JP, Fagan AM, Townsend RR, Holtzman DM (2005) Comparative proteomic analysis of intra- and interindividual variation in human cerebrospinal fluid. Mol Cell Proteomics 4: 2000–2009.
61. Amacher DE, Adler R, Herath A, Townsend RR (2005) Use of proteomic methods to identify serum biomarkers associated with rat liver toxicity or hypertrophy. Clin Chem 51: 1796–1803.
62. Marionneau C, LeDuc RD, Rohrs HW, Link AJ, Townsend RR, et al. (2009) Proteomic analyses of native brain K(V)4.2 channel complexes. Channels (Austin) 3: 284–294.
63. Keller A, Eng J, Zhang N, Li XJ, Aebersold R (2005) A uniform proteomics MS/MS analysis platform utilizing open XML file formats. Mol Syst Biol 1: 2005 0017.
64. Nesvizhskii AI, Keller A, Kolker E, Aebersold R (2003) A statistical model for identifying proteins by tandem mass spectrometry. Anal Chem 75: 4646–4658.
